# Single-cell RNA sequencing of terminal ileal biopsies identifies signatures of Crohn’s disease pathogenesis

**DOI:** 10.1038/s41588-026-02634-7

**Published:** 2026-06-15

**Authors:** Monika Krzak, Tobi Alegbe, D. Leland Taylor, Gareth-Rhys Jones, Mennatallah Ghouraba, Michelle Strickland, Bradley T. Harris, Reem Satti, Kenneth Arestang, Lucia Ramirez-Navarro, Nilanga Nishad, Kimberly Ai Xian Cheam, Marcus Tutert, Matiss Ozols, Guillaume Noell, Steven Leonard, Moritz J. Przybilla, Ciro Ramirez Suastegui, Eleonora Khabirova, Tong Deng, Hanna Najgebauer, Velislava Petrova, Carla P. Jones, Noor Wana, May Xueqi Hu, Jason Skelton, Jasmin Ostermayer, Yong Gu, Wendy Garri, Biljana Brezina, Charry Queen Caballes, Daniele Corridoni, Miles Parkes, Vivek Iyer, Cristina Cotobal Martin, Rebecca E. McIntyre, Tim Raine, Carl A. Anderson

**Affiliations:** 1 Wellcome Sanger Institute, Hinxton, UK; 2 Open Targets, Hinxton, UK; 3 Centre for Inflammation Research, Queens Medical Research institute, University of Edinburgh, Edinburgh, UK; 4 Department of Gastroenterology, Addenbrooke’s Hospital, Cambridge University Hospitals, Cambridge, UK; 5 Sanofi R&D, Cambridge, UK

**Keywords:** Gene expression, Crohn's disease

## Abstract

Crohn’s disease (CD) is a chronic inflammatory bowel disease exhibiting substantial heterogeneity in clinical presentation and response to therapy. To explore its molecular basis, we developed IBDverse, a large single-cell RNA sequencing (scRNA-seq) dataset of terminal ileal biopsies, profiling over 1.1 million cells from 111 patients with CD and 232 healthy controls. This resource integrates discovery and replication cohorts for the robust identification of CD-associated cell types, genes and pathways. We uncovered epithelial changes marked by interferon-driven upregulation of major histocompatibility complex class I molecules that persisted in progenitor cells after macroscopic inflammation resolution. *ITGA4*^+^ macrophages were identified as key inflammatory drivers, showing enriched JAK–STAT signaling and cytokine expression (interleukin-6 (IL-6), IL-12 and IL-23). Heritability analysis linked inflammatory monocytes and macrophages to CD susceptibility, implicating resident and recruited immune cells in pathogenesis. These findings establish a comprehensive cellular and molecular framework for CD, offering insights into disease mechanisms and therapeutic opportunities.

## Abstract (web summary)

IBDverse is a single-cell RNA atlas of terminal ileal biopsies comprising 1.1 million cells from 111 patients with Crohn’s disease and 232 healthy participants. Analysis of the data identifies genes, pathways and cell populations associated with the disease and implicates inflammatory monocytes and macrophages in disease susceptibility.

## Main

Crohn’s disease (CD) is a debilitating inflammatory bowel disease (IBD) characterized by chronic relapsing and remitting inflammation of the gastrointestinal tract. Although inflammation is most commonly observed in the terminal ileum, CD exhibits substantial heterogeneity in disease location, severity and behavior, both between patients and within patients, over time. Although therapies targeting cytokines such as tumor necrosis factor (TNF), interleukin-12 (IL-12) and IL-23 have improved clinical outcomes, primary non-response and secondary loss of response to treatment remains high, with 15% of patients with CD requiring surgical intervention within five years of diagnosis^1,2^. Consequently, there is an urgent need to better understand the etiology of CD in order to broaden therapeutic opportunities.

Single-cell RNA sequencing (scRNA-seq) technologies provide a high-throughput means to dissect complex tissues at the resolution of single cells and cell types. scRNA-seq atlases of the gastrointestinal tract have already facilitated some important discoveries, including the pathogenic remodeling of mesenchymal cells^3^ and *CD8*^+^ T cells^4^ in ulcerative colitis (another common form of IBD), as well as the identification of *BEST4*^+^ enterocytes, which are crucial in maintaining luminal pH in both ulcerative colitis and CD^5–7^, and the identification of metaplastic cells in IBD^8^. However, existing studies have been constrained by small sample sizes, limiting both statistical power and reproducibility due to disease heterogeneity and technical noise.

To address this, we present IBDverse, an scRNA-seq data resource of over 1.1 million cells from terminal ileal biopsies of 111 patients with CD and 232 healthy controls. Using these data and a split-cohort study design, we identify and replicate genes that are aberrantly expressed in CD, plus those where expression is specific to given cell types and cellular processes. We then identify which of these cell types and processes are likely to play a causal role in disease by quantifying their enrichment within IBD genetic association signals.

## Results

### Atlassing terminal ileal biopsies identifies 57 cell clusters

**Fig. 1:**
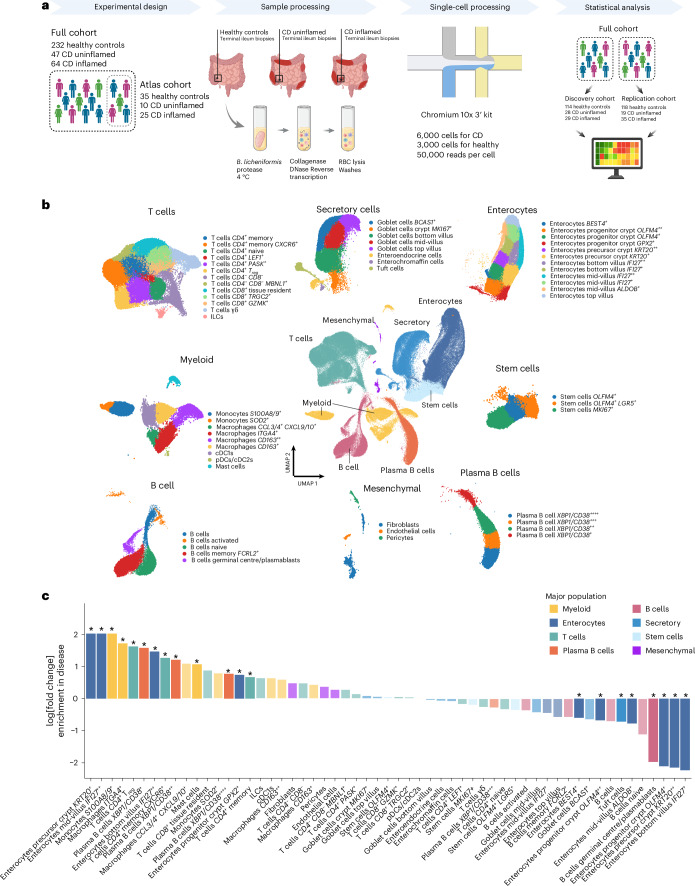
Single-cell expression atlas for the terminal ileum in healthy controls and patients with CD. **a**, Terminal ileum biopsies from 343 individuals were dissociated to single cells on ice in HBSS containing *B. licheniformis* protease. This was followed by a brief incubation in collagenase and then red blood cell (RBC) lysis buffer. Single-cell suspensions were then profiled using a Chromium 10x 3′ kit (Methods). Samples from the full cohort were assigned at random to either the discovery or replication cohort and all reported analyses are required to be statistically significant in both cohorts. **b**, Center, uniform manifold approximation and projection (UMAP) of ~216,000 cells from the atlas cohort that met quality control criteria (Methods), with eight colors representing the primary cell populations. Surrounding the central UMAP, these major populations are further subdivided into 57 distinct cell subtypes. **c**, Differential cell-type abundance between healthy controls and participants with CD for the full cohort. Values show the log[fold change] for CD versus healthy controls. Positive values indicate enrichment in CD and negative values indicate enrichment in healthy controls. Asterisks denote cell types significantly and reproducibly (same direction and FDR < 0.05 in both cohorts) enriched in either health or disease. cDC1s, conventional type 1 dendritic cells; ILCs, innate lymphoid cells; pDCs, plasmacytoid dendritic cells; T_reg_ cell, regulatory T cell. Panel **a** created in BioRender: **a**, Krzak, M. https://biorender.com/wyrw6nl (2026).

Terminal ileal biopsies were profiled from 343 individuals (232 healthy controls and 111 patients with CD) (Fig. 1a). Patients with CD were significantly younger than controls (mean ages of 41 and 49 years, respectively) but did not differ in sex or smoking status (Table 1). The CD biopsies were classified using a score based on three inflammatory components (inflamed surface, ulcer size and ulcerated surface) of the validated simple endoscopic score for CD (SES-CD)^9^, applied to the terminal ileal segment via a single central reader (TI-SES-CD score). This resulted in 64 inflamed (TI-SES-CD ≥ 3) and 47 uninflamed (TI-SES-CD < 3) biopsies from 111 individuals.

**Table 1:** Demographics of healthy samples and those from patients with CD in the IBDverse dataset

Demographic	CD	Healthy	*P* value
*n*	111	232	
Inflammation = minimal or none (%)	47 (42.3)	232 (100.0)	<2.2 × 10^−16^
Sex = female (%)	63 (56.8)	114 (49.1)	0.205
Smoking status (%)			0.208
Never	77 (69.4)	178 (76.7)	
Yes	21 (18.9)	25 (10.8)	
Ex-smoker	12 (10.8)	25 (10.8)	
Vape only	1 (0.9)	4 (1.7)	
Mean age (s.d.)	40.87 (12.12)	49.27 (13.31)	2.00 × 10^−8^
Mean genes (s.d.)	2,315.21 (633.58)	2,445.45 (643.99)	7.78 × 10^−2^
Mean UMIs (s.d.)	12,105.25 (4,105.60)	11,946.12 (4,300.81)	0.741

Inflammation was determined based on the TI-SES-CD, an aggregated score quantifying the degree of inflammation in the terminal ileum. Patients were stratified into two groups: inflamed (TI-SES-CD ≥ 3) and minimal or no inflammation (TI-SES-CD < 3). Significant differences (*P* < 0.05) were determined by two-sided Fisher’s exact test (categorical variables) or two-sided *t*-test (numeric variables).

A representative cell-atlassing cohort was derived from 70 randomly selected samples (35 from individuals with CD and 35 from controls), identifying 57 unique cell clusters across epithelial (enterocytes, secretory cells and stem cells), immune (T, B, plasma B and myeloid cells) and mesenchymal compartments (Fig. 1b and Supplementary Fig. 1). Marker genes for each of the 57 unique cell clusters were identified using Wilcoxon rank-sum tests, and manual annotations were performed by integrating literature references and lineage-defining gene expression profiles (Supplementary Fig. 2, Supplementary Table 1 and Methods). Positional marker genes^10^ were employed to annotate enterocytes and secretory cells along the crypt–villus axis (Supplementary Fig. 3). A cell-type classifier was trained on the clusters from the atlassing cohort and used to annotate the remaining 273 samples, as well as the atlassing cohort. The classifier permitted the inclusion of cells that did not meet the stringent quality control criteria for atlassing but could be confidently identified (Supplementary Fig. 4). Within the atlassing cohort, comparison between classifier-assigned labels and manually annotated labels showed strong concordance, with over half of the cells in 49 of the clusters matching perfectly (Supplementary Fig. 5). Reassuringly, misannotation of cells was often between transcriptomically similar cells, probably reflecting the weakness in applying cut-offs to continuous biology, rather than a failure of the classifier. We also calculated specifically expressed genes in both the original and auto-annotated clusters and saw a high correlation (Pearson’s *R* > 0.77; Supplementary Fig. 6 and Methods). To ensure that our labels matched existing resources, we applied our classifier to re-annotate the clusters from a comparable, publicly available single-cell ileum dataset^11^ and found that the majority of labels agreed (Supplementary Table 2).

To directly assess the replicability of our analyses and address the claims that single-cell sequencing is facing a reproducibility crisis^12–14^, all 343 samples in the study were randomly allocated to either a discovery or replication cohort (Fig. 1a). No significant differences were observed in baseline demographics between the two cohorts (Supplementary Table 3). The resulting single-cell analysis datasets comprised 611,992 cells in the discovery cohort and 573,869 cells in the replication cohort that passed quality control (Methods).

Thirteen cell types were significantly enriched in CD across both cohorts (false discovery rate (FDR) < 5%; log[fold change] > 0), including several that are known to accumulate in intestinal inflammation (Fig. 1c). For example, *S100A8/9*^+^ monocytes and *CXCL9/CXCL10*^+^ macrophages were effectively absent (<2%) in the healthy myeloid compartment (Supplementary Fig. 7). In disease, these cell types comprised 25 and 7%, respectively, of all myeloid cells, in keeping with their known role in pathogen and inflammatory response^15–18^. We also identified CD-specific enterocytes (*GPX2*^+^ progenitor cells and *IFI27*^+^ villus cells; Supplementary Fig. 7) displaying transcriptional signatures of barrier response and antimicrobial activity. For example, both the bottom and middle *IFI27*^++^ villus clusters were denoted by high expression of *CEACAM20* (encoding a sensor of Gram-negative bacteria and driver of IL-8 release through SAP-1 phosphorylation), antimicrobial peptides (encoded by *REG1/3B*) and cytokine responsive elements (encoded by *NOS2* and *HSD3B2*), although expression of these markers was consistently lower in the middle versus bottom villus cluster. *HSD3B2*, encoding a steroid synthetase that is essential for progesterone production, has been shown to modulate local cytokine and repair mechanisms, highlighting the epithelium as not simply a physical barrier, but a key component of the inflammatory cascade, including extra-adrenal steroid production^19,20^.

### Dysregulated gene expression in CD

We performed mixed-model differential gene expression (DGE) analysis between inflamed CD samples and uninflamed healthy samples within each of the discovery, replication and full (both cohorts combined) cohorts. We found 4,241 unique differentially expressed genes (DEGs) in the discovery cohort, 4,166 DEGs in the replication cohort and 5,385 DEGs in the full cohort (Supplementary Table 4; FDR < 5%). The number of DEGs was positively correlated (*R*_discovery_ = 0.66; *R*_replication_ = 0.52; *R*_full_ = 0.73) with the number of sequenced cells (Supplementary Fig. 8a), albeit with variation at the level of individual cell types (Supplementary Fig. 8b). Secretory epithelial cells, enterocytes and T cells had the highest numbers of DEGs in the full cohort (6,527, 6,324 and 4,866, respectively), whereas plasma B cells had only 719 DEGs, despite the large number of sequenced cells (>70,000 in the full cohort).

**Fig. 2:**
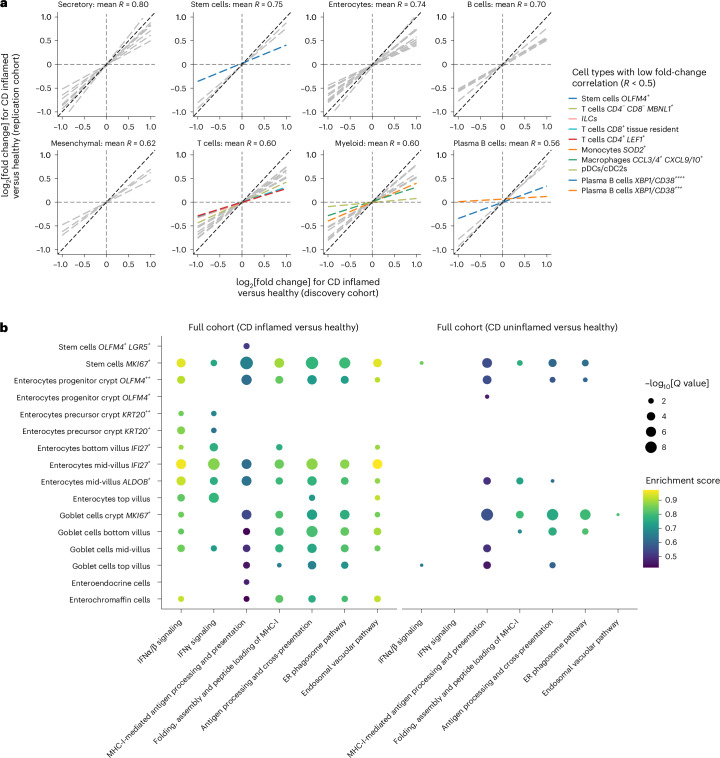
Replicable DGE signatures in CD epithelial cells enriched for IFN signaling and MHC-I antigen presentation. **a**, Linear regression (dashed lines) between log_2_[fold changes] of DEGs (without thresholding, as outlined in the Methods) in the discovery (*x* axis) and replication (*y* axis) datasets. The reported mean *R* values represent the average of regression coefficients calculated across cell types within each major cell population. Cell types with low fold-change correlation (Pearson *R* < 0.5; Supplementary Table 5) are highlighted. **b**, GSEA was performed on DGE *z* scores derived from the full cohort, comparing CD inflamed samples (*n* = 64) versus controls (*n* = 232) and CD uninflamed samples (*n* = 47) versus controls (*n* = 232). The results are shown for epithelial cell types with a high fold-change correlation (Pearson *R* ≥ 0.5).

Only 44% of DEGs detected in the discovery cohort were also significantly dysregulated (FDR < 5%) in the same cell type, with the same direction of effect, in the replication cohort (Supplementary Fig. 8b). Correlation of fold-change estimates between the two cohorts revealed that epithelial cell DEGs were the most replicable (mean *R* ≥ 0.74; Fig. 2a) and plasma B cell DEGs were the least replicable (mean *R* = 0.56). However, all major populations showed wide variation in fold-change replicability between constituent cell types. For example, fold-change estimates were more consistent for γδ T cells (*R* = 0.82) than for *LEF1*^+^ T cells (*R* = 0.28) or *CD8*^+^ tissue-resident T cells (*R* = 0.32) (Supplementary Table 5). There was also significant variation in fold-change correlation between individual cell types in the myeloid compartment, with *ITGA4*^+^ macrophages demonstrating the best agreement (*R* = 0.88) and plasmacytoid dendritic cells and type 2 conventional dendritic cells the least (*R* = 0.12). Four of the ten cell types with a fold-change correlation of <0.5 (plasmacytoid dendritic cells, type 2 conventional dendritic cells, tissue-resident *CD8*^+^ T cells, *OLFM4*^+^ stem cells and *XBP1/CD38*^+++^ and *XBP1/CD38*^++++^ plasma B cells) showed poor annotation consistency between the manual and auto-annotation approaches in the atlassing cohort (fewer than 50% of cells concordantly annotated). Two myeloid cell types (*SOD2*^+^ monocytes and *CCL3/4*^+^*CXCL9/10*^+^ macrophages) showed poor fold-change correlations (*R* < 0.5) despite high consistency between the manual and auto-annotation (>98% of cells concordantly annotated). For these cell types, poor replicability was probably underpinned by imbalance in the number of case and control cells. Fold-change replicability patterns were not improved by using a pseudobulk differential expression approach (Supplementary Fig. 9).

### Pan-epithelial and cell-type-specific changes in active CD

We undertook gene set enrichment analysis (GSEA) on the 47 cell types with the greatest replicability in our CD inflamed versus healthy DEG analysis (Supplementary Table 5; threshold *R* ≥ 0.5).

We observed upregulation of interferon α/β (IFNα/β) signaling in 12 epithelial cell types along the entire crypt–villus axis (Fig. 2b). Leading-edge genes, namely *IFI27*, *HLA-A/B/C/E/F*, *IFITM3*, *PSMB8*, *STAT1* and *IRF1*, were differentially expressed across all 12 cell types (Supplementary Table 6). Many of these genes also featured in other significantly enriched pan-epithelial pathways, including upregulation of: antigen processing and cross-presentation; folding, assembly and peptide loading of major histocompatibility complex class I (MHC-I); the ER phagosome pathway; and the endosomal vacuolar pathway. Although MHC-I pathways were upregulated across many cell types, the epithelial cell types were over-represented, as 22 of the top 25 enrichments (by enrichment score) were epithelial. We also compared biopsies from patients with uninflamed CD with those of healthy controls and did not observe an enrichment of DEGs in the IFNα/β pathway, except for weak but statistically significant enrichment in *MKI67*^+^ stem cells. This suggests that the enrichment of type I IFN signaling across epithelial cell types in active CD is a widespread barrier response to injury that largely subsides after repair.

Many of the genes differentially expressed between cells from patients with inflamed CD versus those from healthy biopsies were enriched in the folding, assembly and peptide loading of MHC-I pathway, including *CANX*, *CALR*, *TAPBP* and *B2M*, across 12 individual epithelial cell types representing all major subtypes (Supplementary Table 6). However, type II IFN responses—through the IFNγ signaling pathway—appeared more restricted, with enrichment preferentially to enterocytes along the entire crypt–villus axis, but not in other epithelial cell types (for example, secretory cells) (Fig. 2b). Given that multiple MHC-I pathways and associated genes were consistently enriched across epithelial cell types, we tested whether inflammation severity (TI-SES-CD score) was associated with the strength of this effect. The pathways MHC-I-mediated antigen processing and presentation; folding, assembly and peptide loading of MHC-I; and antigen processing and cross-presentation remained enriched in all epithelial cell types except the top-villus goblet cell and enteroendocrine subsets (Supplementary Fig. 10a). This suggests a widespread dose-dependent effect of inflammation on MHC-I upregulation. Importantly, analyses comparing biopsies from patients with non-inflamed CD with those from healthy controls showed that this upregulation of MHC-I antigen presentation persisted even after the resolution of inflammation (Fig. 2c).

To validate our findings, we generated scRNA-seq data from intestinal organoids that were either left unstimulated or treated with IFNγ to emulate the inflamed intestinal state (Methods). Twelve epithelial cell types were annotated with high confidence in both stimulated and unstimulated organoids (Supplementary Fig. 11), enabling paired comparisons of stimulation-induced transcriptional changes. Consistent with our primary tissue analyses, IFNγ stimulation induced upregulation of the IFNγ signaling and MHC-I antigen processing pathways across the majority of epithelial cell types (Supplementary Fig. 10b), supporting an epithelial-intrinsic antigen presentation response in inflammation. We also performed a comparison between health and disease in the unstimulated organoids and, in line with the biopsy data, observed weak enrichment of selected MHC-I pathways (Supplementary Fig. 10b), indicating that some elements of the antigen presentation program persist outside the inflamed micro-environment.

Although MHC-I is expressed by all cells, MHC-II expression is thought to be restricted to professional antigen-presenting cells to limit activation of the adaptive immune system through T cell receptor engagement. However, in the context of inflammation, intestinal epithelial cells (IECs) have been shown to upregulate MHC-II molecules^5,15,21,22^. Consistent with this, we also observed upregulation of some MHC-II signaling genes in our ileal samples (*HLA-DRA*, *HLA-DRB1*, *HLA-DPA1*, *HLA-DRB5*, *HLA-DQB1* and *HLA-DMB*), but only within specific enterocyte and stem cell subsets (mid-villus *ALDOB*^+^, mid-villus *IFI27*^+^, progenitor crypt *OLFM4*^++^ and *MKI67*^+^ stem cells) (Supplementary Fig. 10c). Thus MHC-II signaling upregulation was less widespread across epithelial cells compared with that of MHC-I.

Collectively, these results suggest widespread changes across epithelial subtypes relating to type I IFN and MHC-I signaling, with these changes associated with inflammation severity. Importantly, these perturbations in MHC-I signaling may persist in key progenitor cells long after the initial inflammatory stimuli have been removed. This is consistent with studies of pancreatic β cells in type 1 diabetes mellitus, whereby single exposure to type 1 IFN results in long-lasting MHC-I overexpression and has also recently been shown to reprogram the MHC-I repertoire towards an HLA-B bias in these cells^23,24^. Alongside these changes in immune potential in epithelial cells, we also detected evidence of shifts in metabolic function with evidence of alterations in pathways and genes associated with both oxidative phosphorylation and glycolysis (Supplementary Fig. 10d).

### *ITGA4*^+^ macrophages are enriched for JAK–STAT signaling

**Fig. 3:**
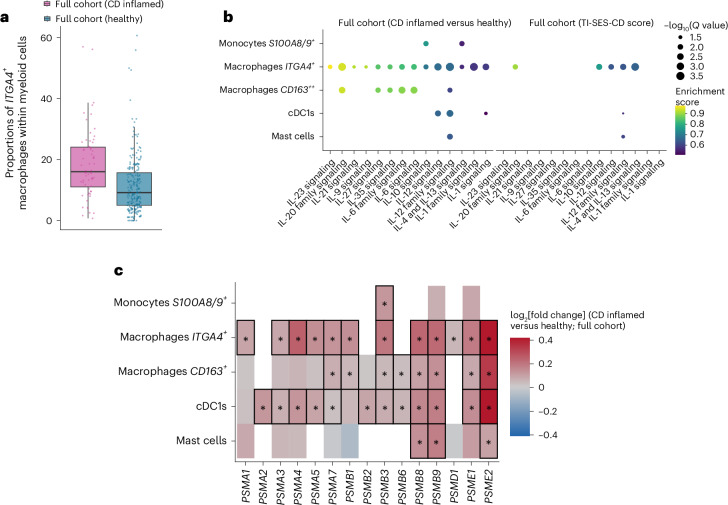
*ITGA4*^+^ macrophages upregulate cytokine and proteasome genes during inflammation. **a**, Proportions of *ITGA4*^+^ macrophages within myeloid cells across samples from participants with inflamed CD (*n* = 64) and healthy controls (*n* = 232). The central lines show median values, the box bounds show the second and third quartile values and the whiskers show 1.5× the interquartile range. **b**, GSEA of DGE between samples from participants with inflamed CD (*n* = 64) and healthy controls (*n* = 232) and across CD samples with varying severity of inflammation (*n* = 111). CD samples were stratified based on the TI-SES-CD, with scores ranging from 0–9. Enrichment is shown for replicable myeloid cell types (Pearson *R* ≥ 0.5; Supplementary Table 5). **c**, Fold change and significance (FDR < 0.05; asterisks) of proteasome and immunoproteasome genes differentially expressed in the replicable myeloid cell populations.

Genetic variants that increase *ITGA4* (CD49d) expression in stimulated monocytes are associated with increased IBD risk. *ITGA4*, which is targeted by the IBD therapy vedolizumab, was most highly expressed in two myeloid populations: *ITGA4*^+^ macrophages and *CD163*^++^ macrophages (Supplementary Fig. 2). *ITGA4*^+^ macrophages were significantly expanded in inflamed CD (averaging 16% of myeloid cells versus 10% in health; Fig. 3a and Supplementary Fig. 12a) and showed the highest DGE replicability (*R* = 0.88) (Supplementary Table 5). GSEA revealed cytokine involvement across an array of interleukin pathways (Fig. 3b). These cytokines suggest a predominant role of signaling through receptors of the IL-6 and IFN superfamilies, mainly mediated by Janus kinase 1 (JAK1), JAK2 and tyrosine kinase 2 (refs. ^25,26^). Concordantly, *JAK2* and *STAT1* were the most overexpressed genes, alongside negative regulators of JAK–signal transducers and activators of transcription (STAT) signaling, such as *PTPN1*/*2*. Expression levels of *IL10*, *IL12* and *IL20* were also positively associated with inflammation severity (TI-SES-CD score) in *ITGA4*^+^ macrophages. *ITGA4*^+^ macrophages also upregulated genes associated with the complex formation of proteasomes (for example, *PSMA4/5*) and immunoproteasomes (for example, *PSMB8/9*)—processes known to be dependent on IFN signaling (Fig. 3c)—suggesting that they play a role in MHC-I-mediated presentation to *CD8*^+^ T cells.

Taken together, our findings show that *ITGA4*^+^ macrophages are over-represented in CD inflammation and preferentially express an array of cytokine signaling pathways. The specific repertoire of interleukins suggests IL-6 and IFN super-family receptor signaling with resultant JAK activation, particularly that of JAK2, with inflammation-dependent increases in *IL10*, *IL12* and *IL20*. These cells further demonstrate the effects of type II IFN signaling, with enrichment for many pathways involved in immunoproteasome–MHC-I communication, suggesting an active role in cytokine sensing, signaling and adaptive immune cell cross-talk.

### IBD genetics acts across gut cell populations

**Fig. 4:**
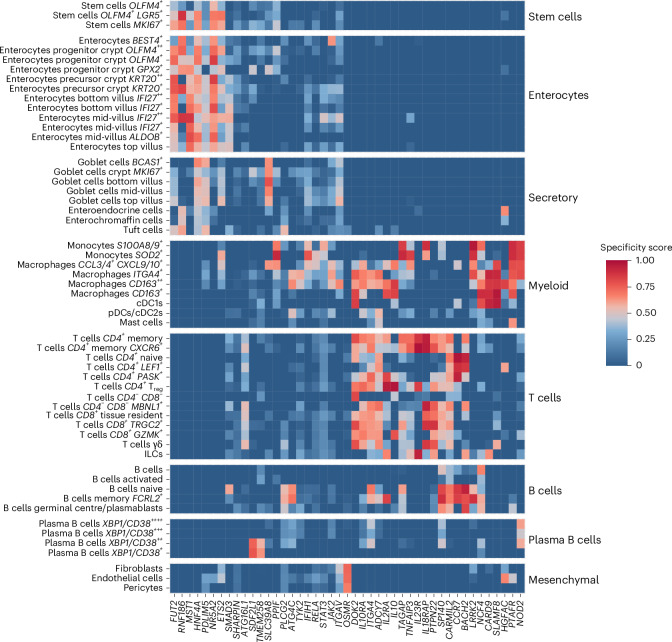
IBD risk genes show lineage-specific expression across terminal ileum cell types. Specificity of IBD risk gene expression across 57 terminal ileum cell types in the full cohort (*n* = 343; CD and healthy samples). For prioritization criteria, see Methods.

Genome-wide association studies (GWAS) have identified more than 300 regions of the human genome associated with IBD susceptibility. We compared gene-expression specificity scores across the 57 cell types in our atlas for 45 high-confidence candidate effector genes (Methods) from IBD GWASs (Fig. 4). All eight major cell populations showed specific expression of at least one IBD effector gene, highlighting the complex cellular architecture of IBD. *FUT2*, a gene that when disrupted leads to dysbiosis and increased susceptibility to inflammation^27,28^, was specifically expressed by multiple enterocyte and stem cell populations (specificity > 0.50) compared with other major cell populations (specificity < 0.36). Specific expression was greatest in precursors and progenitor cells and fell progressively as enterocytes matured up the crypt.

*NOD2* mutations comprise the strongest genetic effects on ileal CD in Western populations and have been suggested to impair the handling of intracellular bacteria by myeloid cells^29–31^. We observed specific expression of *NOD2* in monocytes (*S100A8/9*^+^ and *SOD2*^+^) and immature macrophages (for example, *ITGA4*^+^ and *CXCL9/10*^+^) (mean specificity = 0.8), but not resident macrophages (for example, *CD163*^+^ and *CD163*^++^) (mean specificity = 0.2). Across all of these cell types, we found no evidence of *NOD2* differential expression between biopsies from patients with inflamed CD and those from healthy individuals (Supplementary Table 4). Three of these *NOD2*^+^ cell types (*SOD2*^+^ monocytes, *S100A8/9*^+^ monocytes and *CXCL9/10*^+^ macrophages) were also the sole source of oncostatin M (*OSM*) in our data (mean specificity = 0.9). OSM has been proposed as an IBD biomarker and therapeutic target, inducing a stromal inflammatory program (including *IL6*, *CXCL9* and *CXCL10*), with high pretreatment levels associated with anti-TNF failure^32^. The gene encoding the cognate receptor, *OSMR*, is expressed by CD45^−^EpCAM^−^CD31^−^ cells and its expression is co-linear with that of *COL1A1*, *FAP* and *PDPN* fibroblast markers^32^ at the whole-tissue level. In our study, we confirm cell-specific expression of *OSMR* within fibroblast, endothelial and pericyte subpopulations of mesenchymal cells (specificity = 0.74, 0.72 and 0.67, respectively).

**Fig. 5:**
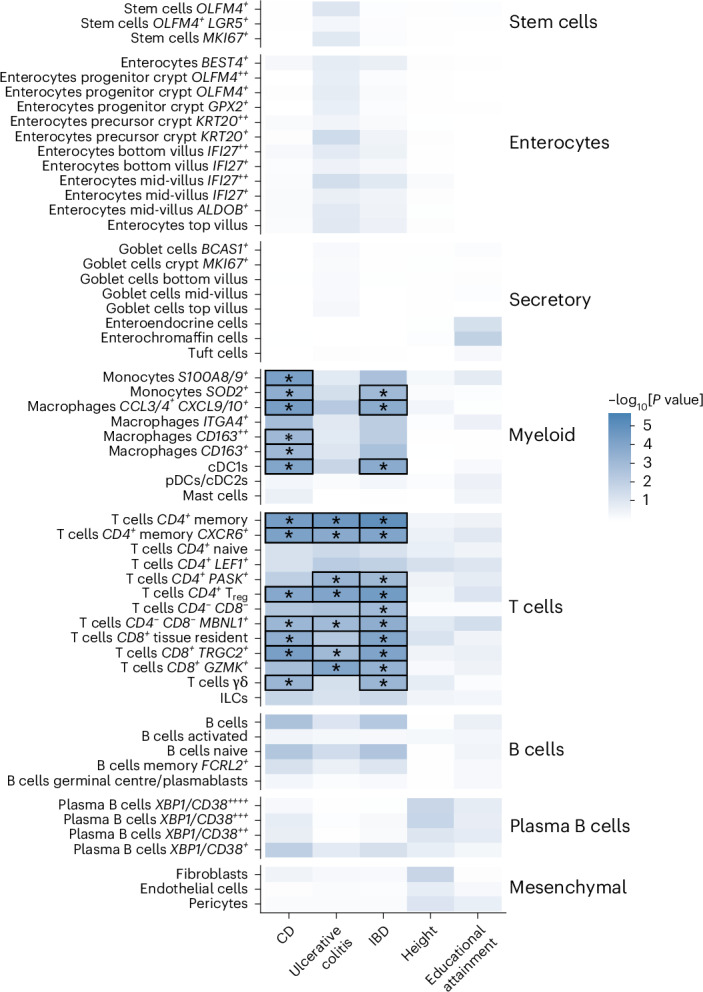
Myeloid and T cells are enriched for disease heritability. Enrichment of CD, ulcerative colitis and IBD heritability in functional annotations based on specifically expressed genes of each cell type in the full cohort. Enrichment was tested using stratified linkage disequilibrium score regression. *P* values were obtained from a one-sided test for a positive effect and multiple comparisons were controlled at a family-wise error rate of <0.05 using the Bonferroni procedure across all tested cell types. Significance (asterisks) was consistent for the same analysis performed in both the discovery and replication cohorts. Height and educational attainment were used as negative controls.

To identify cell types of pathological relevance beyond individual loci, we correlated cell-type-specific gene expression scores with genome-wide maps of genetic susceptibility for CD, ulcerative colitis and IBD, using height and educational attainment as negative controls^33–36^. Only immune cell types showed enrichment of heritability for CD, ulcerative colitis and IBD. Among innate immune cells, specifically expressed genes within *S100A8/9*^+^ and *SOD2*^+^ monocytes, *CXCL9/CXCL10*^+^ macrophages, *CD163*^+/++^ cells and conventional type 1 dendritic cells were significantly enriched for CD heritability (Fig. 5; family-wise error rate < 5%). This suggests a potential causal role for cell types involved in both inflammatory responses (*S100A8/9*^+^ monocytes and *CXCL9/10*^+^ macrophages) and immune tolerance (*CD163*^+^ macrophages and conventional type 1 dendritic cells) in CD pathogenesis. Furthermore, myeloid populations with genes significantly enriched for CD heritability, including *S100A8/9*^+^ monocytes, *SOD2*^+^ monocytes and *CCL3/4*^+^*CXCL9/10*^+^ macrophages, were significantly reduced in healthy samples (Supplementary Fig. 12; FDR < 5%). Within the adaptive immune compartment, genes specifically expressed by *CD4*^+^*CXCR6*^+^ memory T cells, regulatory T cells, *TRGC2*^+^*CD8*^+^ T cells and *CD4*^*−*^*CD8*^*−*^ T cells were enriched for both CD and ulcerative colitis heritability, whereas *CD8*^+^ tissue-resident T cells and γδ T cells were specifically enriched in CD (Fig. 5; family-wise error rate < 5%).

## Discussion

We established IBDverse, one of the largest scRNA-seq cohorts of terminal ileum biopsies, with discovery and replication cohorts designed to address reproducibility challenges in single-cell genomics. Despite rigorous standardization of biopsy collection, processing and analysis, fewer than 50% of DEGs replicated between cohorts. Replicability varied markedly across cell types, with poorly annotated or rare populations exhibiting particularly low replication rates. For instance, the scarcity of *S100A8/9*^+^ monocytes and *CXCL9/CXCL10*^+^ macrophages in healthy biopsies limited our ability to reliably detect DEGs. Larger studies are essential to improve the detection of DEGs in these disease-relevant but underrepresented cell types.

Additionally, the variable transcriptional plasticity of immune cells—shaped by factors such as diet, inflammation severity, disease stage and treatment regimens—probably contributes to low replication rates. Our findings underscore the challenges of conducting scRNA-seq differential gene-expression studies on complex, heterogeneous patient populations and highlight the need for caution, particularly when interpreting unreplicated findings from small-scale DGE studies.

Our analyses identified a consistent barrier response in the intestinal epithelium, centered on type I and II IFN signaling. Genes involved in MHC-I function and type I IFN pathways were upregulated across the crypt–villus axis. This pan-epithelial MHC-I response to barrier damage was largely absent in other cell types and persisted even after resolution of macroscopic inflammation. These findings suggest that inflammation leaves a molecular scar within epithelial progenitor cells that persists after the resolution of inflammation and probably influences responses to future insults. This aligns with recent work by Dennison et al.^37^ in pediatric IBD showing that loss of DNA methylation at MHC-I loci, including *NLRC5*, enhances MHC-I gene expression in epithelial organoids. We demonstrate that MHC-I expression remains elevated after inflammation in adult patients, particularly in stem-like progenitor cells.

The persistent elevation of MHC-I expression following resolution of macroscopic inflammation is likely to have significant implications for barrier function, perhaps via non-canonical antigen presentation by IECs^38^. Exogenous antigens can access late endosomal compartments in IECs and colocalize with MHC-I proteins in patients with CD, raising the possibility of cross-presentation. This process may enable IECs to present luminal antigens to CD8^+^ T cells, highlighting a potential role for epithelial cells in shaping immune responses. This phenomenon of cross-presentation of exogenous antigens by non-professional antigen-presenting cells has been reported in murine renal epithelium^39^ and liver endothelium^40^, and in the context of the intestine suggests that epithelial cells play an active role in shaping local inflammatory responses that may be dysregulated in CD.

Epithelial MHC-I expression driven by IFN signaling has been observed in various tissues and disease contexts. For instance, during murine *Citrobacter* species infection, IFN**γ** sensing in the intestinal epithelium facilitates both pathogen and self-antigen presentation to intraepithelial T cells via interferon regulatory factor 1 and MHC-I, which suppresses NLRP3 macrophage inflammasome activation. Notably, mice with transgenic deletion of IFNγ sensing (Ifngr^fl/fl^VilCre) exhibit decreased interferon regulatory factor 1–MHC-I signaling and resistance to anti-TNF therapy^41^. Building on these findings, our data demonstrate that increased epithelial type II IFN responses are predominantly restricted to enterocytes along the crypt–villus axis and resolve following tissue repair.

Macrophages are among the most abundant leukocytes in the gastrointestinal tract and play a critical role in intestinal health^42^. Their dysregulation is strongly associated with chronic inflammatory diseases such as IBD^42,43^. During active disease, intestinal monocytes and their macrophage progeny accumulate in large numbers, producing pro-inflammatory cytokines such as TNF, IL-1β, IL-6, and IL-23 (refs. ^44–46^). In this study, we identified a transcriptionally distinct population of *ITGA4*^+^ macrophages that become more abundant during intestinal inflammation. DEGs in these cells are enriched in nine cytokine pathways, including IL-6, IL-12 and IL-23. This response is underpinned by a *JAK*/*STAT* gene signature, with *JAK2* and *STAT1/2/3* being significantly differentially expressed. Given that JAK inhibitors, which target multiple cytokine-dependent pathways (for example, IL-6, IL-10 and IL-23), have therapeutic benefit in IBD, understanding their cellular targets is critical. Importantly, macrophages from patients with IBD carrying disease-associated polymorphisms in *JAK2* display enhanced *JAK2* expression and NOD2-induced JAK2 phosphorylation and amplified cytokine signaling^47^. Moreover, *JAK2*-deficient macrophages fail to upregulate MHC-I proteins in response to type I IFN stimulation, increasing susceptibility to infection and inflammation^48,49^. We identify *ITGA4*^+^ macrophages as a preferential source of JAK–STAT signaling and downstream cytokine responses in CD, highlighting their potential as therapeutic targets.

The strong enrichment of CD heritability among *CXCL9/10*^+^ macrophages and *SOD2*^+^ and *S100A8*/*9*^+^ monocytes is noteworthy given that both populations are significantly expanded in disease and highly express inflammatory cytokines plus known CD susceptibility genes such as *NOD2*, *PTAFR* and *LRRK2*. Unfortunately, we were unable to detect many reproducible DEGs in these cell types, probably due to their rarity in healthy biopsies. We note that although we did not observe heritability enrichment in non-immune cells, this has been reported previously (for example, within M cells in ulcerative colitis^50^). Our data show that several probable effector genes within IBD-associated loci are specifically expressed by non-immune cells, including *RNF186* (ref. ^51^), *FUT2* (ref. ^52^), *PDLIM5* (ref. ^53^) and *HNF4A*^54^. The underrepresentation of stromal cells in our atlas due to a combination of using pinch biopsies and digestive enzymes that favor epithelial cell capture probably also reduces our power to detect enrichments.

In summary, we present IBDverse, one of the largest scRNA-seq datasets of terminal ileal biopsies, comprising over 1.1 million cells from 111 patients with CD and 232 healthy controls. Using discovery and replication cohorts, we have identified and validated genes, pathways and cell types associated with CD. We have found widespread epithelial changes driven by IFN signaling and persistent MHC-I upregulation following inflammation, as well as a distinct population of *ITGA4*-expressing macrophages that are major contributors to JAK–STAT signaling and cytokine production. Heritability analysis highlighted the involvement of immune cell populations, such as *CXCL9/10*^+^ macrophages and *S100A8/9*^+^ monocytes, in CD pathogenesis. These data and results, accessible at https://www.ibdverse.info/, provide a valuable resource for understanding CD mechanisms and identifying potential therapeutic targets.

## Methods

### Sample ascertainment

This study was approved by the National Health Service Research Ethics Committee (Cambridge South; REC ID 17/EE/0338). Written informed consent was given by all participants.

Individuals undergoing routine endoscopic assessment were recruited at Addenbrooke’s hospital, Cambridge, UK. All participants with CD classified as inflamed had a confirmed history of CD and macroscopic evidence of terminal ileal inflammation from tissue sampled during the biopsy. All control participants were undergoing endoscopic assessment or surveillance for healthy and non-cancer-related reasons (for example, a history of iron deficiency anemia or a family history of colorectal cancer). Control participants did not have macroscopic evidence of intestinal inflammation or a personal history of cancer and were not in receipt of corticosteroids or any other immune-modulating therapy. Pinch biopsies of the terminal ileum were collected from all participants and deposited into pre-chilled Hanks balanced salt solution (HBSS) without Mg^2+^, Ca^2+^ or phenol red (HBSS^−/−^). Samples were placed on ice and immediately transferred to the Sanger Institute.

### Single-cell RNA isolation and sequencing

Terminal ileal biopsies were dissociated on ice to release all major intestinal cell types present in the biopsy (epithelial, immune and stromal) without stressing the cells and to minimize de novo transcription. Two to three pinch biopsies were transferred to HBSS^−/−^ containing 2 mM ethylenediaminetetraacetic acid (EDTA), 0.26 U µl^−1^ serine endoprotease isolated from *Bacillus licheniformis* (P5380; Sigma–Aldrich), 5 µM QVD-OPh (ab141421; Abcam) and 50 µM Y-27632 dihydrochloride (ab120129; Abcam). The biopsies were mechanically minced and gently pipetted every 10 min during a 30-min incubation period on ice. Cells were washed with 1% bovine serum albumin (BSA) HBSS^−/−^, centrifuged at 350*g* at 4 °C for 5 min and then incubated for 10 min at room temperature in HBSS with Mg^2+^ and Ca^2+^ and without phenol red—including 5 mM CaCl_2_, 1.5 U µl^−1^ collagenase IV (LS004188; Worthington Biochemical) and 0.1 mg ml^−1^ DNase I (07900; Stem Cell Technologies). Next, 0.5 M EDTA was added and the cells were filtered (30 µm; 04-0042-2316; CellTrics), washed and centrifuged before being incubated for 3 min at room temperature in red blood cell lysis buffer (ACK lysing buffer; A10492; Gibco). Cells were washed and centrifuged twice before a final filtration (40 µm; 352340; Falcon) and manual cell counting (by hemocytometer; DHC-N01; NanoEnTek). Step-by-step detailed protocol can be found at ref. ^55^.

For some samples, we isolated the cells released in the buffer after mechanical mincing (fraction 1) and mixed them at different ratios with cells isolated from the remaining tissue chunks after enzymatic digestion (fraction 2) in an attempt to make the representation of immune cell types more equal to epithelial cells in variably inflamed samples^56^. Fractions 1 and 2 were processed the same way throughout the protocol, except that fraction 1 did not undergo the first serine endoprotease enzymatic digestion step.

Single-cell RNA-seq was undertaken using 3′ 10x Genomics kits (versions 3.0 and 3.1) according to the manufacturer’s instructions. All samples sequenced under kit version 3.1 had dual indexes, whereas samples sequenced under kit version version 3.0 had either single or dual indexes. We targeted 6,000 cells for participants with CD and 3,000 cells for controls to account for the increased cellular heterogeneity in CD biopsies. The viability of the mixed populations was 92 ± 9% (mean ± s.d.) according to trypan blue staining. Libraries were sequenced using a HiSeq 4000 sequencer (Illumina; *n*_CD_ = 20; *n*_control_ = 4) or NovaSeq S4 Xp sequencer (Illumina; *n*_CD_ = 91; *n*_control _= 228) with 100-base pair paired-end reads, targeting 50,000 reads per cell. We compared the fraction of reads mapped confidently to the transcriptome (the output metric from CellRanger) within participants with CD and healthy controls and found no difference between sequencers (minimum *P* > 0.05; Wilcoxon rank-sum test).

### Mucosal organoid derivation, culture and passages

Fresh or cryopreserved terminal ileum biopsies were obtained with written informed consent from participants (three healthy donors and four individuals with CD). Organoids were derived as follows: biopsies were washed with chilled Dulbecco’s phosphate-buffered saline (D-PBS) containing penicillin–streptomycin (100 U ml^−1^), transferred to a six-well plate with 2.5 ml Dulbecco’s modified Eagle medium (DMEM)/F-12 (11330-032; Thermo Fisher Scientific) and incubated for 30 min at 37 °C under 5% CO_2_. After incubation, biopsies were transferred to a 15 ml tube containing 5 ml 2.5 mM EDTA in D-PBS and gently shaken on ice for 35 min on a rotator. Tissue was allowed to settle and the supernatant was discarded. The tissue was washed with D-PBS, allowed to settle again and the supernatant was discarded. D-PBS was added and the tissue was pipetted up and down with a 1% BSA-coated P1000 tip to further fragment the tissue; pieces were allowed to settle and most of the supernatant was removed. Next, 2 ml 1% BSA in DMEM/F-12 was added and the suspension was pipetted 15 times with a 1% BSA-coated tip to release intestinal crypts. Tissue pieces were allowed to settle and the supernatant was filtered through a 70 µm strainer into a 1% BSA–D-PBS-coated tube. This step was repeated once and the filter was washed with fresh 1% BSA–DMEM/F-12. The filtrate was centrifuged at 800*g* for 5 min and the supernatant was gently removed. Matrigel (356231; Corning) was prepared and mixed with the released crypts by gently pipetting ten times. The mixture was split into two to six domes per biopsy and plated one dome per well on a pre-warmed 48-well plate and incubated at 37 °C until the domes solidified.

Complete crypt culture medium (250 µl; IntestiCult Organoid Growth Medium (Human) (basal (100-0190) plus supplement (100-019)), 1% penicillin–streptomycin (15140122; Gibco) and a 10 μM final concentration of the Rho/ROCK pathway inhibitor Y-27632 dihydrochloride (72307; STEMCELL Technologies)) was then added to each well.

Once established, organoids were passaged every 2 days from passage 1 to passage 7, depending on the sample, following this protocol. The medium was removed and, using a P1000 wide-bore tip, 250 µl cold Cell Recovery Solution (354253; Corning) was added per well. Domes were scraped and transferred to a 50 ml tube, then pipetted up and down five times to break the Matrigel pieces. An additional 250 µl cold Cell Recovery Solution was used to wash each well, with gentle pipetting using a wide-bore tip to retrieve the remaining organoids. The suspension was incubated on ice for 18 min with occasional flicking, then spun at 300*g* for 5 min to remove any residual Matrigel. The supernatant was removed, 4 ml chilled 1% BSA in DMEM/F-12 was added and the sample was spun at 300*g* for 3 min at 4 °C. After removing the supernatant, Matrigel was added at a ratio of 20 µl per well of a 48-well plate. The organoid/Matrigel mixture was pipetted up and down ten times to mix without introducing bubbles and 20 µl domes were plated into a 48-well plate. Plates were incubated at 37 °C for at least 10 min to solidify the Matrigel, then 250 µl complete crypt culture medium was added. The medium was changed every 2 days and passaging was performed every 7–10 days.

### Stimulation with IFNγ and single-cell dissociation for 3′ 10x scRNA-seq

Selected organoid lines were differentiated and two cultures per line were maintained to compare unstimulated versus stimulated conditions by adding human IFNγ recombinant protein (10 µg ml^−1^; 300-02-100UG; PeproTech) to the culture medium for 5 days to mimic chronic inflammation. Organoids were then collected and processed for single-cell dissociation as follows.

Organoids from three wells per condition were collected. Cell Recovery Solution (200 µl) was added to each well, Matrigel was scraped using a wide-bore tip and the mixture was pooled into a 1.5 ml tube on ice. Tubes were kept on ice for 18 min, inverted every 2 min, then centrifuged at 300*g* for 3 min at 4 °C to pellet the cells, after which supernatant was removed. Residual Matrigel was washed off twice by adding 500 µl wash buffer (HBSS^−/−^ + 1% BSA + 2 mM EDTA) and centrifuging at 350*g* for 4 min at 4 °C.

The pellet was resuspended in residual buffer by flicking the tube, then 500 µl pre-warmed TrypLE enzyme (12563011; Gibco) was added. Using a pre-wetted P1000 tip, the suspension was pipette mixed to break up the organoids. The tubes were incubated at 37 °C for 15 min until dissociation was complete, after which 500 µl cold wash buffer was added and the samples were centrifuged at 300*g* for 3 min at 4 °C to pellet the cells. ACK lysis was then performed by adding 500 µl ACK lysing buffer (A1049201; Thermo Fisher Scientific), incubating at room temperature for 3 min, topping up with 500 µl wash buffer and centrifuging at 300*g* for 3 min at 4 °C. The supernatant was poured off and the cells were resuspended in residual buffer, topped up with wash buffer and centrifuged again at 300*g* for 3 min at 4 °C. This wash was repeated once more, followed by a final top up with HBSS^−/−^ + 1% BSA and centrifugation at 300*g* for 3 min at 4 °C. After the final spin, the supernatant was removed, the pellet was resuspended in D-PBS with a pre-wetted P200 tip and the suspension was filtered through a 40 µm strainer. The cells were counted under a microscope using trypan blue to assess viability and concentration.

Cells from three organoid lines were pooled, and approximately 9,000 cells were targeted for scRNA-seq using the 3′ 10x Genomics v3 chemistry, following the manufacturer’s instructions.

### Single-cell RNA-seq processing and quality control procedures

CellRanger version 7.2.0 was used to demultiplex reads, align reads to GRCh38 with Ensembl version 93 transcript definitions (GRCh38-3.0.0 reference file distributed by 10x Genomics) and generate cell-by-gene-count matrices. CellBender version 2.1 (ref. ^57^) was then applied to identify droplets containing cells and adjust the raw counts matrix for background ambient transcript contamination. For training, CellBender requires a rough estimate of the number of droplets containing cells (cell droplets) and the number of droplets without cells (empty droplets) derived from the unique molecular identifier (UMI) curve (that is, the rank ordering of droplet barcodes according to total UMI counts (*x* axis) by the total number of UMI counts per droplet (*y* axis)). The UMI curve was calculated from droplets with a UMI count of >1,000 and the threshold was estimated using the barcoderanks-inflection procedure from DropletUtils version 1.9.16 (ref. ^58^). To estimate the number of empty droplets, we calculated the UMI curve as described above, selected droplets with a UMI count of between 250 and 10 and estimated the threshold by performing both the barcoderanks-inflection and barcoderanks-knee procedures from DropletUtils, using one-third of the distance between the two estimates as the final threshold. CellBender was run with default parameters except for excluding droplets with fewer than ten UMI counts (--low-count-threshold) and using 300 epochs with a learning rate of 1 × 10^−7^. The final counts matrix was adjusted for the ambient transcript signature at a false positive rate of 0.1. Next, multiplets were identified and removed using scrublet version 0.2.1 (ref. ^59^), simulating 100,000 multiplets and calculating the multiplet threshold using the threshold_li function from scikit-image package version 0.17.2 (ref. ^60^), initialized using the threshold_otsu function. The reported sex of each sample was verified by generating pseudobulk expression matrices and comparing the expression of *XIST* with the mean expression of all genes on the Y chromosome.

### De novo cell-type identification

The atlassing cohort was used to identify cell types and fit a model to automatically predict cell types across the entire dataset. First, additional filters were applied to ensure only the highest-quality cells were used for de novo clustering. Cells with fewer than 100 genes expressed at ≥1 count, or where the percentage of counts originating from the mitochondrial genome (https://www.genenames.org/data/genegroup/#!/group/1972) was >50, were removed. Next, an isolation forest (scikit-learn version 0.23.2) was used to remove outlier cells based on: (1) the percentage of counts originating from the mitochondrial genome; (2) the total number of UMI counts per cell; and (3) the number of genes expressed (≥1 count) per cell. These metrics were selected following the recommendations in ref. ^61^.

Subsequent processing and management of the expression data was performed using scanpy version 1.6.0 (ref. ^62^). Genes expressed (≥1 count) in five or fewer cells across the whole dataset were removed (sc.pp.filter_genes with min_cells = 5). To account for variable sequencing depths across cells, UMI counts were normalized by the total number of counts per cell and scaled to counts per 10,000 (CP10K; sc.pp.normalise_per_cell) and the CP10K expression matrix (ln[CP10K + 1]; sc.pp.log1p) was log transformed.

To perform dimensionality reduction, the 2,000 most variable genes across samples were selected by: (1) calculating the most variable genes per sample; and (2) selecting the 2,000 genes that occurred most often across samples (sc.pp.highly_variable_genes with flavor = ‘seurat’ and batch_key = sample). After mean centering and scaling the ln[CP10K + 1] expression matrix to unit variance, principal component analysis (sc.tl.pca) was undertaken using the 2,000 most variable genes after the removal of protein coding mitochondrial, ribosomal and immunoglobulin genes, because these genes constituted the ambient signature learned by CellBender. To select the number of principal components for subsequent analyses, we used a scree plot^63^ and calculated the knee/elbow derived from the variance explained by each principal component using kneedle estimator version 0.7.0 (ref. ^64^). From the automatically estimated elbow, we included five additional principal components to ensure that all meaningful variability was captured, selecting 29 principal components for clustering. Finally, bbknn version 1.3.12 (ref. ^65^) was applied to integrate samples and control for sample-specific batch effects.

Clusters were defined using the Leiden graph-based clustering algorithm version 0.8.3 (ref. ^66^) on the nearest neighbors determined by bbknn. Clusters were generated across a range of resolutions from 0.5–5.0 to empirically determine the optimal clustering resolution. For each resolution considered, the data were divided into training (two-thirds of cells) and test sets (one-third of cells) and a single-layer dense neural network was fit to predict cluster identity from expression using keras version 2.4.3. The cluster label of each cell was predicted and the Matthews correlation coefficient (MCC) was calculated for each cluster^67^. The final cluster classifications were chosen to achieve a minimum MCC of > 0.75 across all clusters, with a resolution of 3.25 selected to meet this criterion (Supplementary Fig. 13). At this resolution, all clusters met the threshold except for cluster 40, which exhibited MCC < 0.75 at many resolutions and was therefore excluded. This adjustment yielded a total of 57 clusters.

### Cell-type annotation

To determine the cell-type identity of the 57 clusters, marker genes for each cluster were identified using the Wilcoxon rank-sum test (sc.tl.rank_genes_groups with method = ‘wilcoxon’) to compare the gene expression of each cell type with that of all other cell types and rank genes according to differences in expression. Highly discriminative marker genes with a Bonferroni-corrected *P* < 0.05 were then used to label cell types through expert knowledge. To further visualize the annotated cell types, dimensionality reduction was undertaken using the uniform manifold approximation and projection algorithm, implemented within scanpy (scanpy.tl.umap) with default parameters, except for changing the minimum distance from 0.5–1.0. Analyzing all cells that passed quality control, we identified eight major cell populations, including epithelial cells (stem cells, enterocytes and secretory cells), immune cells (T and B cells, plasma B cells and myeloid cells) and mesenchymal cells.

Within epithelial cells, we identified three distinct stem cell populations: *OLFM4*^+^ stem cells, *OLFM4*^+^*LGR5*^+^ stem cells and proliferating *MKI67*^+^ stem cells. Among enterocytes, we identified progenitor cells marked by *OLFM4* and *GPX2*, precursor cells expressing *KRT20* and a range of enterocytes expressing *IFI27*. We further distinguished enterocytes along the crypt–villus axis (crypt, middle and top) based on signature genes such as *ALPI*, *APOA4* and *APOC3* (ref. ^10^). In the secretory cell lineage, we identified goblet cells (*CLCA1*, *FCGBP* and *MUC2*), including proliferating *MKI67*^+^ and *BCAS1*^+^ goblet cells, as well as goblet cells positioned along the crypt–villus axis, marked by *EGFR*, *KLF4*, *NT5E* and *SLC17A5*. Additional cell types included enteroendocrine cells (*NTS*, *PYY* and *GCG*), enterochromaffin cells (*TPH1* and *CES1*) and tuft cells (*PLCG2*, *PTGS1* and *LRMP*). A summary of these markers and their expression across cell types is visualized in Supplementary Fig. 2.

Within immune cells, we identified monocytes expressing *S100A8/9*, *SOD2* and *CXCL9/10*, macrophages with positive expression of *ITGA4*, resident macrophage populations (*CD163*, *MAF* and *C1QA/B/C*), type 1 conventional dendritic cells (*XCR1* and *BATF3*), a mix of plasmacytoid dendritic cells and type 2 conventional dendritic cells (determined by *IRF4*, *ZEB1* and *FLT3*) and mast cells (*MS4A2* and *TPSAB1*) (Supplementary Fig. 2). Additionally, we identified 13 distinct T cell populations, including *CD4*^+^ and *CD8*^+^ T cells, innate lymphoid cells (marked by *IL1R1*, *ALDOC* and *LSTI*) and γδ T cells (*TRGC1*, *TRDC* and *GZMA/B*). Within the *CD4*^+^ T cell subset, we characterized naive T cells (*SELL* and *CCR7*), *LEF1*^+^- and *PASK*^+^-expressing *CD4*^+^ T cells, regulatory T cells (marked by *FOXP3* and *TIGIT*) and two populations of double-negative (*CD4*^−^*CD8*^−^) T cells, one of which showed elevated *MBLN1* expression. In the *CD8*^+^ T cell subset, we identified two populations of tissue-resident *CD8*^+^ T cells expressing *TRGC2*, as well as a distinct population of GZMK^+^-expressing *CD8*^+^ T cells (Supplementary Fig. 2).

Among B cells, we identified *FAU*-expressing B cells, activated B cells (*CKS1B* and *STMN1*), naive B cells (*IGHD/M* and *FCER2*) and germinal center and plasmablast B cells (*CD19*, *CD38* and *TCL1A*). Additionally, we observed a gradient of plasma cells with varying levels of *XBP1* and *CD38* expression (Supplementary Fig. 2).

Lastly, we identified three mesenchymal cell populations: fibroblasts (*COL1A1/2* and *COL3A1*), endothelial cells (*PECAM1* and *VWF*) expressing *ACKR1* and pericytes (*PDGFRB* and *CSPG4*) (Supplementary Fig. 2).

We did not detect a distinct group of neutrophil and eosinophil cells. As has previously been well documented, they are poorly represented due to the limited ability of the 10x scRNA-seq process to capture granulocytes^68^.

### Crypt–villus score

We positioned epithelial cells along the crypt-–villus axis, identifying top-villus epithelial cells as well as enterocyte precursors and progenitor cells and goblet cells located at the crypt base. Stem cells and enterocytes were scored based on the expression of genes such as *APOA4*, *APOC3*, *ALPI*, *PKIB*, *PMP22* and *SLC28A2*, derived from spatial transcriptomics data that characterize crypt and villus intestinal cells^10^. Similarly, we applied the signature genes *EGFR*, *KLF4*, *NT5E* and *SLC17A5* to score secretory cells across the crypt–villus axis.

### Automatic cell-type annotation

To annotate cell types across all samples, we used CellTypist version 1.6.2 (ref. ^69^) to train a classification model based on our identified clusters. This trained CelltTypist model was then applied to assign cell-type labels to all 343 scRNA-seq samples, following the initial processing steps detailed in the section ‘Single-cell RNA-seq processing and quality control procedures’. Cells with a CellTypist confidence score of <0.5 were excluded from subsequent analyses.

### DGE analysis

For each cell type, we tested for the association of gene expression with CD disease status or inflammation severity (TI-SES-CD score) using MAST version 1.14.0, a two-part generalized linear model with a logistic regression component for the discrete process (that is, whether a gene is expressed or not) and a linear regression component for the continuous process (that is, the expression level)^70^. For gene *i*, individual *j* and cell *k*, let *Z*_*ki*_ indicate whether gene *i* is expressed in cell *k* and *Y*_*ki*_ denote the ln[CP10K + 1] normalized gene expression. A two-part regression model was used to test for association:

1 $$\mathrm{logit}\left(P \left({Z}_{{ki}}=1{{|X}}_{k}\right)\right)={X}_{k}{{\boldsymbol{\beta }}}_{i}+{W}_{k}{{\boldsymbol{\gamma }}}_{j}$$

2 $$P \left({Y}_{{ki}}={y|}{Z}_{{ki}}=1\right)=N\left({X}_{k}{{\boldsymbol{\beta }}}_{i}+{W}_{k}{{\boldsymbol{\gamma }}}_{j},\,{\sigma }_{i}^{2}\right)$$

where *X*_*k*_ are the predictor variables for cell *k*, *W*_*k*_ is the random-effect design matrix of cell *k* belonging to individual *j*, **β**_**i**_ is the vector of fixed-effect regression coefficients and **γ**_**j**_ is the vector of random effects (that is, the random complement to **β**_*i*_), normally distributed with mean zero and variance ${\sigma }_{\gamma}^2$. In all DEG comparisons, whether contrasting patients with inflamed or uninflamed CD with healthy controls or examining the association of gene expression with TI-SES-CD score, sex, age (binned into groups of five), cell mitochondrial percentage (a technical covariate associated with cellular stress) and cell complexity (that is, the number of genes detected per cell^6,70^) were included as fixed-effect variables and individual was included as a random effect to control for pseudoreplication bias^71^. The Benjamini–Hochberg procedure^72^ was used to control for multiple testing across all cell types and *P* values were obtained from the hurdle model, derived from the summed *χ*^2^ null distributions of the discrete (*Z*_*i*_) and continuous (*Y*_*i*_) components, as described by Finak et al.^70^. To increase the speed of each test, genes with an average CP10K of <1 in that cell type were removed before fitting models for each cell type. We replicated our single-cell DGE results with a pseudobulk approach using DESeq2 version 1.42.1 (ref. ^73^) and the same model except for the cellular covariates and random effect.

### Differential abundance analysis

Differential abundance analyses were performed by calculating the number of cells present in each cluster for each individual and then transforming counts using the standard limma-voom (limma version 3.42.2) workflow^74^. We used the dream function^75^ to fit a linear model for each cell type to determine the effect of disease status, whereas sex, age and a binary variable for whether the fraction ratio was altered or not were included as fixed covariates. Empirical Bayes shrinkage was applied and *P* values were adjusted for multiple hypothesis testing using the Benjamini–Hochberg procedure across all cell types.

### GSEA

GSEAs were performed using GSEA version 1.17.1 (ref. ^76^) with default parameters to identify pathways enriched among DEGs. Pathways were obtained from the reactome version 76 gene pathway database^77^ as part of MSigDB version 7.4 (ref. ^78^). *Z* scores from the CD versus control DGE hurdle model were used as input for enrichment analyses.

### Prioritization of IBD effector genes

Forty-five genes likely to be perturbed in IBD were identified from within IBD-associated loci based on several criteria, including but not limited to: (1) the presence of a coding mutation fine-mapped down to single-variant resolution; (2) detailed and convincing functional follow-up work that established the causality of the gene; or (3) knowledge that the protein encoded by the gene plays a major role in a pathway that is targeted by an existing IBD therapy. Note that it is typically not straightforward to identify disease effector genes from within GWAS loci and this challenge remains a major focus for the field of complex disease genetics. Although our list of 45 probable IBD effector genes is undoubtedly greatly enriched for true IBD effector genes, false positives could still remain.

### Specifically expressed genes

We identified specifically expressed genes for each cell type using CELLEX package version 1.2.1 (ref. ^34^). CELLEX calculates specifically expressed gene scores using four complementary approaches that include use of the gene enrichment score^79^, expression proportion^80^, normalized specificity index^81^ and differential expression *t*-statistic. The package produces a normalized mean of these four metrics, which we used as our specificity score.

### Heritability analysis

Heritability enrichment analysis was performed using the CELLECT version 1.3.0 workflow^34^. This workflow deploys stratified linkage disequilibrium score regression^33^ to identify cell types with features that are enriched in genetic associations for a disease or trait of interest. CELLECT was run with default parameters, including filtering out complex genetic regions such as the human leukocyte antigen locus before analysis. CELLECT requires summary statistics from a genetic association study and a set of gene scores (ranging between 0 and 1) for each gene that are to be tested for heritability enrichment. For genetic summary statistics of interest, we used IBD, CD and ulcerative colitis statistics^82^, and as negative controls we used genetic summary statistics for height^35^ and educational attainment^36^. For the gene scores, we used the CELLEX cell-type-specific gene scores and applied Bonferroni correction to the resulting *P* values to control for the total number of tests across all cell types.

### Statistics and reproducibility

No statistical method was used to predetermine sample size. Patients who were taking probiotics or antibiotics were excluded to minimize confounding factors. Patients of non-European ancestries and outside the age range of 18–70 years were also excluded. Samples were randomly allocated to two cohorts. There were no significant differences between the two cohorts. Samples were anonymized using unique ID numbers before analyses.

### Reporting summary

Further information on research design is available in the Nature Portfolio Reporting Summary linked to this article.

## Online content

Any methods, additional references, Nature Portfolio reporting summaries, source data, extended data, supplementary information, acknowledgements, peer review information; details of author contributions and competing interests; and statements of data and code availability are available at 10.1038/s41588-026-02634-7.

## Supplementary information

Supplementary Information

Reporting Summary

Peer Review File

Supplementary Tables 1, 2, 5 and 6.

Supplementary Table 4.

## Data Availability

We have centralized all of the data exploration, version history and unrestricted file access on our project website at https://www.ibdverse.info/. We have deposited publicly accessible processed data (single-cell objects, summary statistics, anonymized metadata and models) in the BioStudies database (http://www.ebi.ac.uk/biostudies) under accession numbers S-BSST2944, E-MTAB-16998 and E-MTAB-16999. Raw sequencing data from the ileal samples and clinical metadata are available from the European Genome-phenome Archive (https://ega-archive.org) under accession EGAD00001015692. To protect patient privacy and clinical confidentiality, only this European Genome-phenome Archive dataset is under restricted access. Access will be granted to researchers for related research following approval by the Sanger Data Access Committee.

## Code availability

The code used for analyses within this study is available from GitHub at https://github.com/andersonlab/sc_ti_atlas or via Zenodo at 10.5281/ZENODO.19180889 (ref. ^83^).
