# Supplementary material for: Single-cell RNA sequencing of terminal ileal biopsies identifies signatures of Crohn’s disease pathogenesis
Source: Nat Genet. 2026 Jun 15;58(7):1610–9. doi: 10.1038/s41588-026-02634-7 (PMC13364666; doi:10.1038/s41588-026-02634-7)
Supplement: Supplementary file 1 — Supplementary Information [file 41588_2026_2634_MOESM1_ESM.pdf]

nature genetics

Supplementary information

https://doi.org/10.1038/s41588-026-02634-7

Single-cell RNA sequencing of terminal ileal biopsies identifies signatures of Crohn's disease pathogenesis

In the format provided by the authors and unedited

Supplementary Information

Supplementary Figures

Supplementary Fig. 1. Batch effects in the terminal ileum atlas cohort.

Supplementary Fig. 2. Marker gene expression used to curate annotations within the terminal ileum atlas.

Supplementary Fig. 3. Epithelial cell types represent the crypt-villus axis differentiation.

Supplementary Fig. 4. Quality control metrics.

Supplementary Fig. 5. Accuracy in re-annotating the atlas cohort.

Supplementary Fig. 6. Concordance of gene specificities across discovery and replication datasets.

Supplementary Fig. 7. Cell-type proportions across healthy and CD samples in the atlasing cohort.

Supplementary Fig. 8. Differentially expressed genes between CD inflamed and healthy samples across all 57 cell types.

Supplementary Fig. 9. Replicability of differential gene expression results with pseudobulked samples.

Supplementary Fig. 10. Dysregulated pathways in epithelial cells.

Supplementary Fig. 11. Single-cell expression atlas of ileal-derived organoids stimulated with interferon gamma.

Supplementary Fig. 12. Myeloid cell types enriched for CD heritability are found predominantly in inflamed CD gut biopsies.

Supplementary Fig. 13. Optimisation of cluster resolution for cell-type identification.

Supplementary Tables

Supplementary Table 3. Demographics of samples across cohorts.

Fig. S1. Batch effects in the terminal ileum atlas cohort.

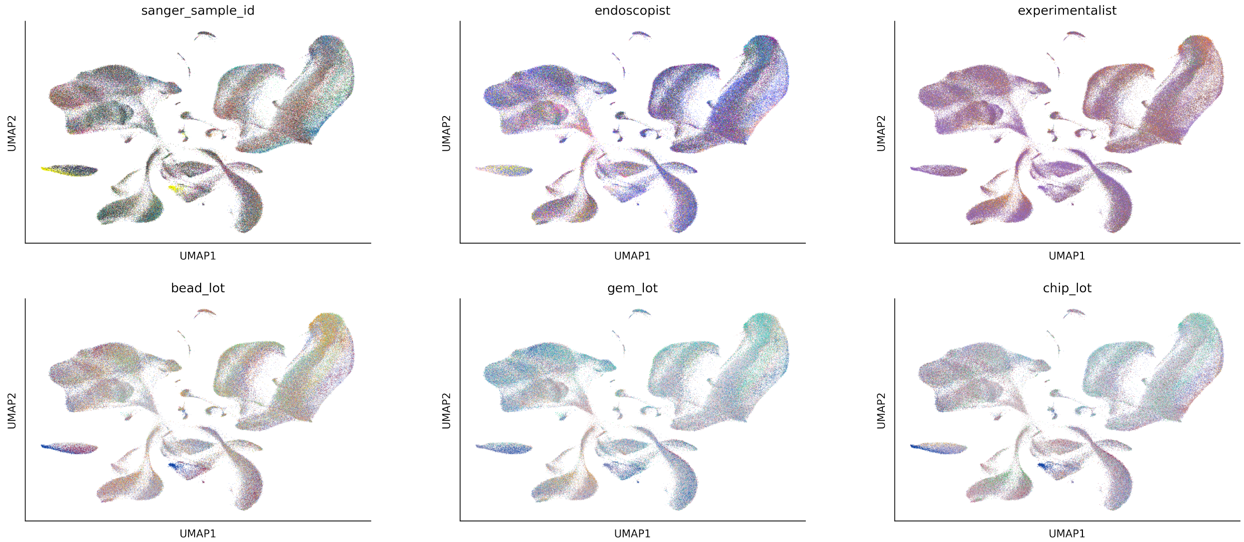

UMAP of the 216k cells from the atlas cohort coloured by potential technical batch effects including the sample ID (the batch-corrected variable, n=70), the endoscopist who took the biopsy (n=31), the experimentalist who processed the sample for single-cell RNA-sequencing (n=8), the 10X bead batch (n=22), the 10x gem batch (n=23) and the 10x chip batch (n=24).

Fig. S2. Marker gene expression used to curate annotations within the terminal ileum atlas.

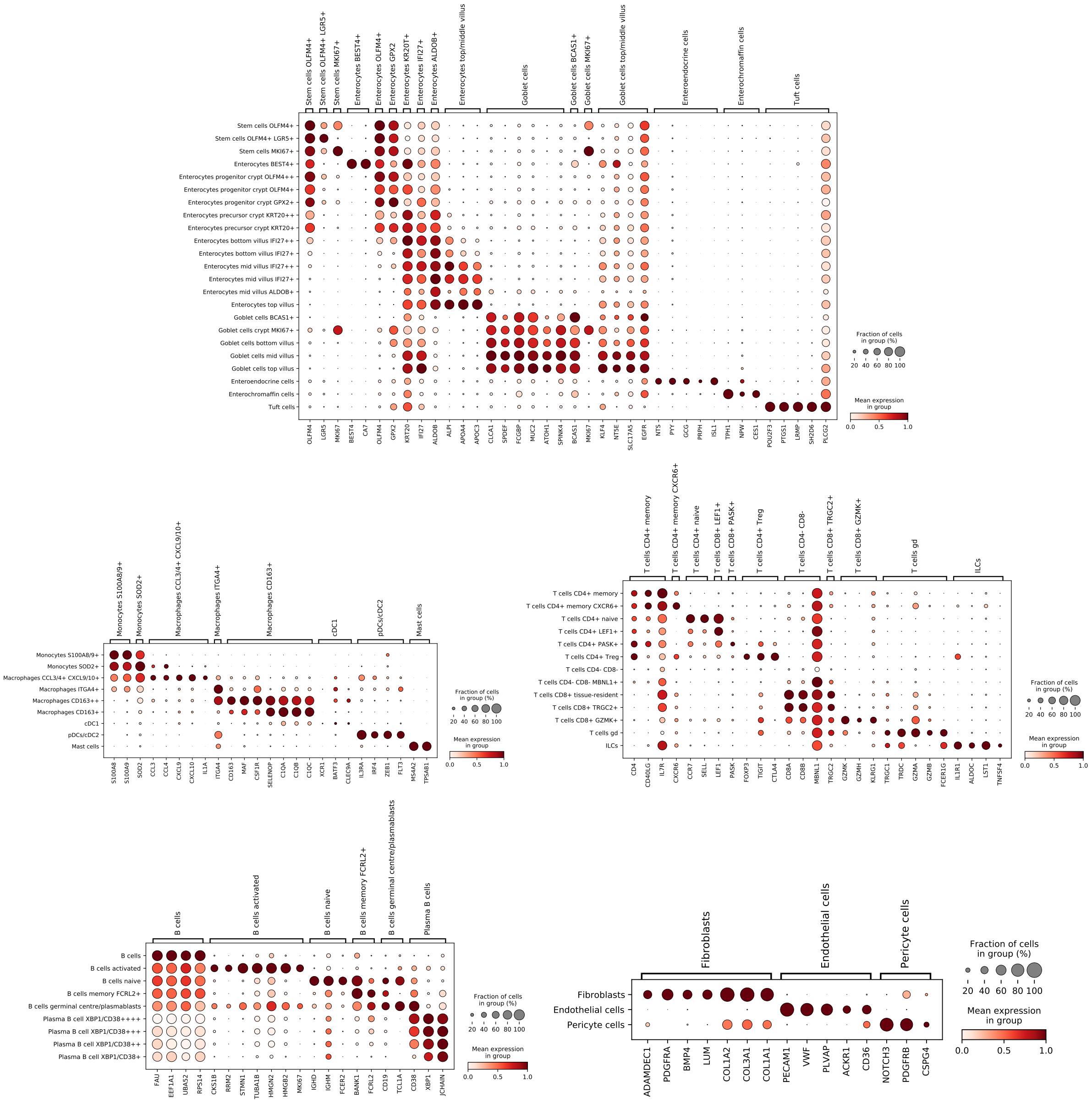

Transcriptional signatures based on literature-derived or expert-curated markers were used to define epithelial, myeloid, T cell, B cell, plasma B cell, and mesenchymal cell types. Dot size indicates the proportion of cells within each cluster expressing a specific marker, color intensity reflects the mean expression of the gene within the cluster.

Fig. S3. Epithelial cell types represent the crypt-villus axis differentiation.

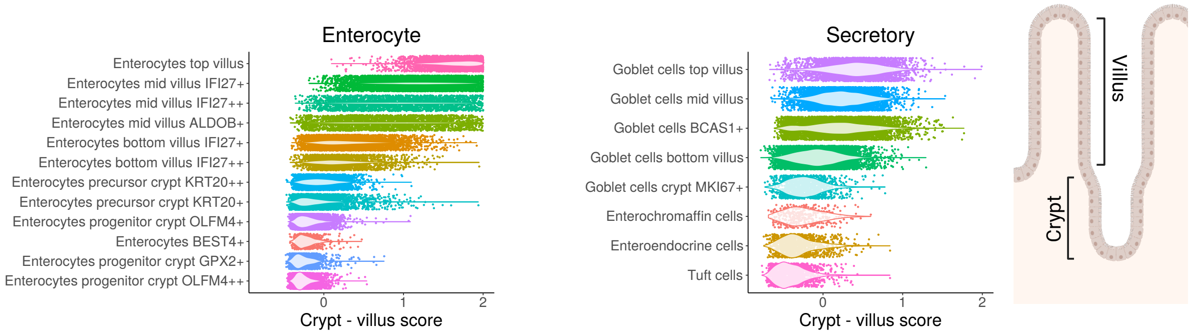

Spatial distribution of enterocytes and secretory cells along the crypt-villus axis was inferred using gene signatures from Moor et al. (2018) (Methods). A subset of enterocytes, termed “top villus enterocytes,” exhibited elevated expression scores for a specific gene signature (including *APOA4, APOC3, ALPI*), indicative of cells located at the villus tip. In contrast, enterocytes situated along the mid-villus or in progenitor/stem cell zones showed lower expression of these markers. Similarly, goblet cells were evaluated for a top-villus gene signature comprising *EGFR, KLF4 and NT5E*. Top villus goblet cells demonstrated slightly higher expression scores compared to goblet cells in mid-villus regions and those at the crypt base. Parts of this figure were created in BioRender. Krzak, M. (2026) https://BioRender.com/xbg8oyk.

Fig. S4. Quality control metrics

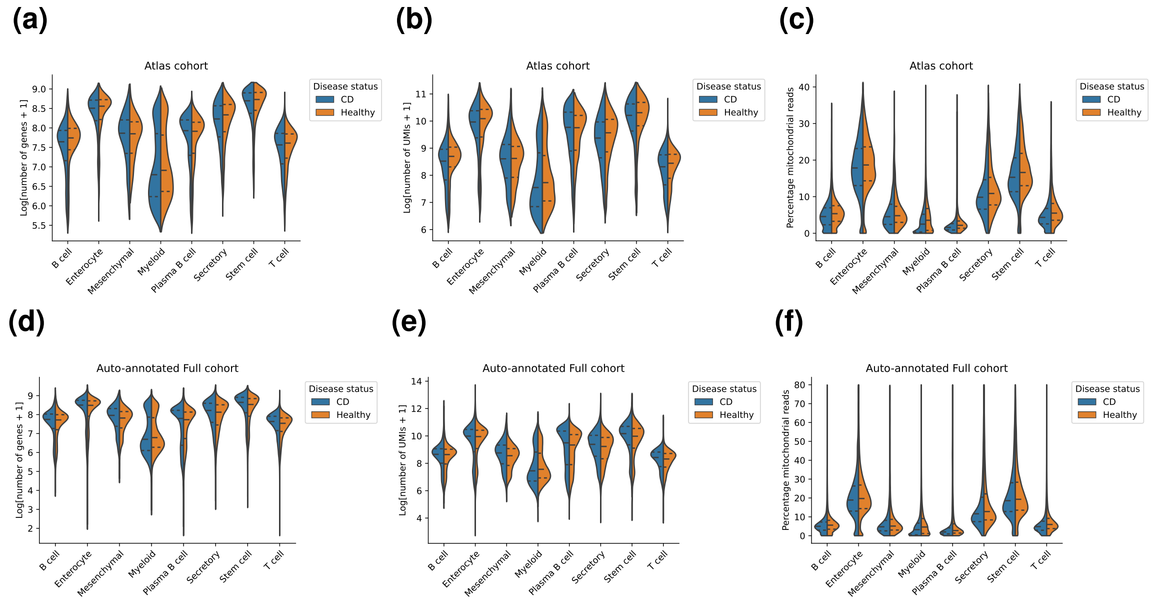

Split violin plots (between Crohn's disease [CD] and healthy controls) of single-cell quality control metrics across the eight major populations captured in the study. Quality control metrics are: Number of genes, number of unique molecular identifiers (UMIs) and percentage of mitochondrial reads per cell across the atlas cohort (n=70, **(a-c)**) full cohort (n=343, **(d-f)**). Lines, from bottom-to-top, within the violins are the first quartile, median and third quartile.

Fig. S5. Accuracy in re-annotating the atlas cohort.

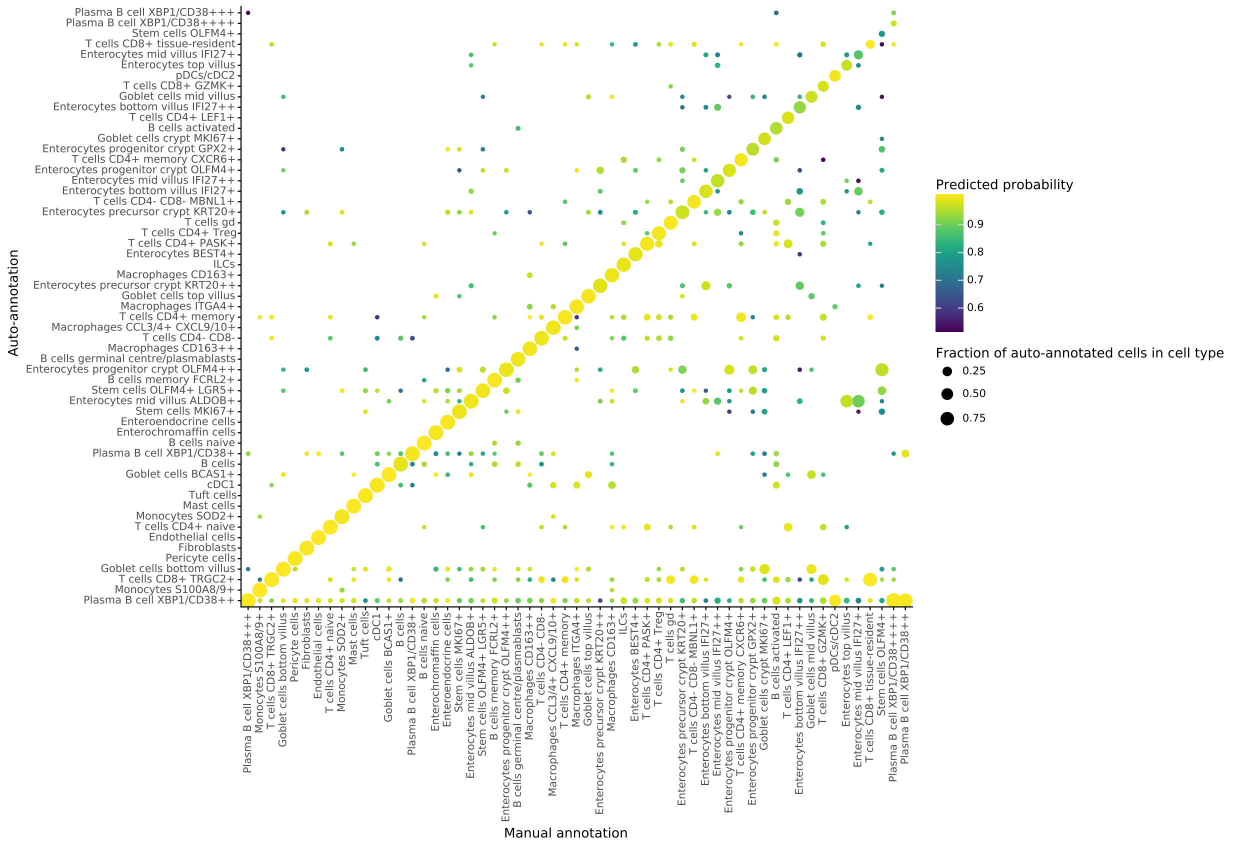

Dot size represents the proportion of auto-annotated cells in the atlas cohort (n=70, y-axis) within each manually defined cluster (x-axis), with color indicating the probability of the mapping prediction.

Fig. S6. Concordance of gene specificities across discovery and replication datasets.

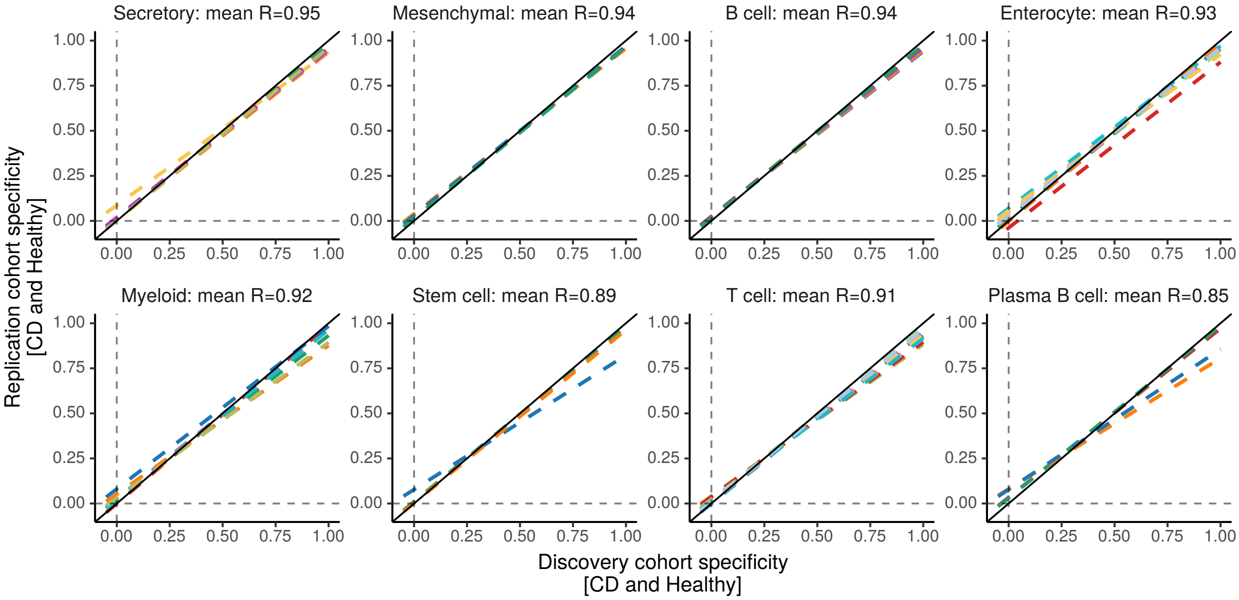

Linear regression model between computationally determined, specifically expressed genes (without thresholding, as outlined in Methods) in the discovery dataset (n=171, x-axis) and replication dataset (n=172, y-axis). For the color legend, please refer to Fig. 1b. The reported mean Pearson R represents the average of regression coefficients calculated across cell types within each major cell population. Both Crohn's disease (CD) and healthy samples were included in this analysis.

Fig. S7. Cell-type proportions across healthy and CD samples in the atlasing cohort.

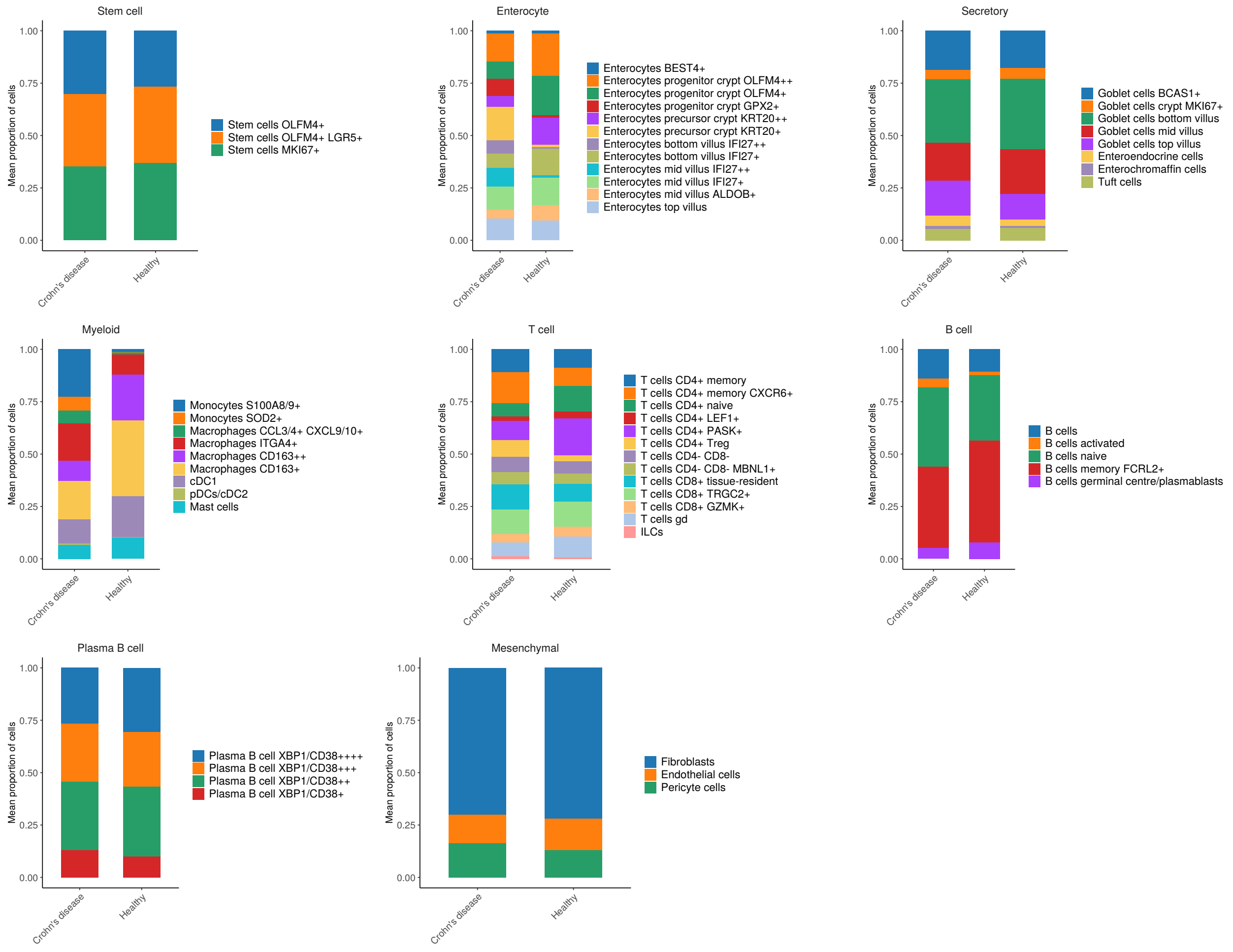

Compositional differences in cell-type proportions between Crohn's disease (CD) patients (n=25) and healthy controls (n=35) across eight major cell populations in the atlas cohort.

Fig. S8. Differentially expressed genes between CD inflamed and healthy samples across all 57 cell types.

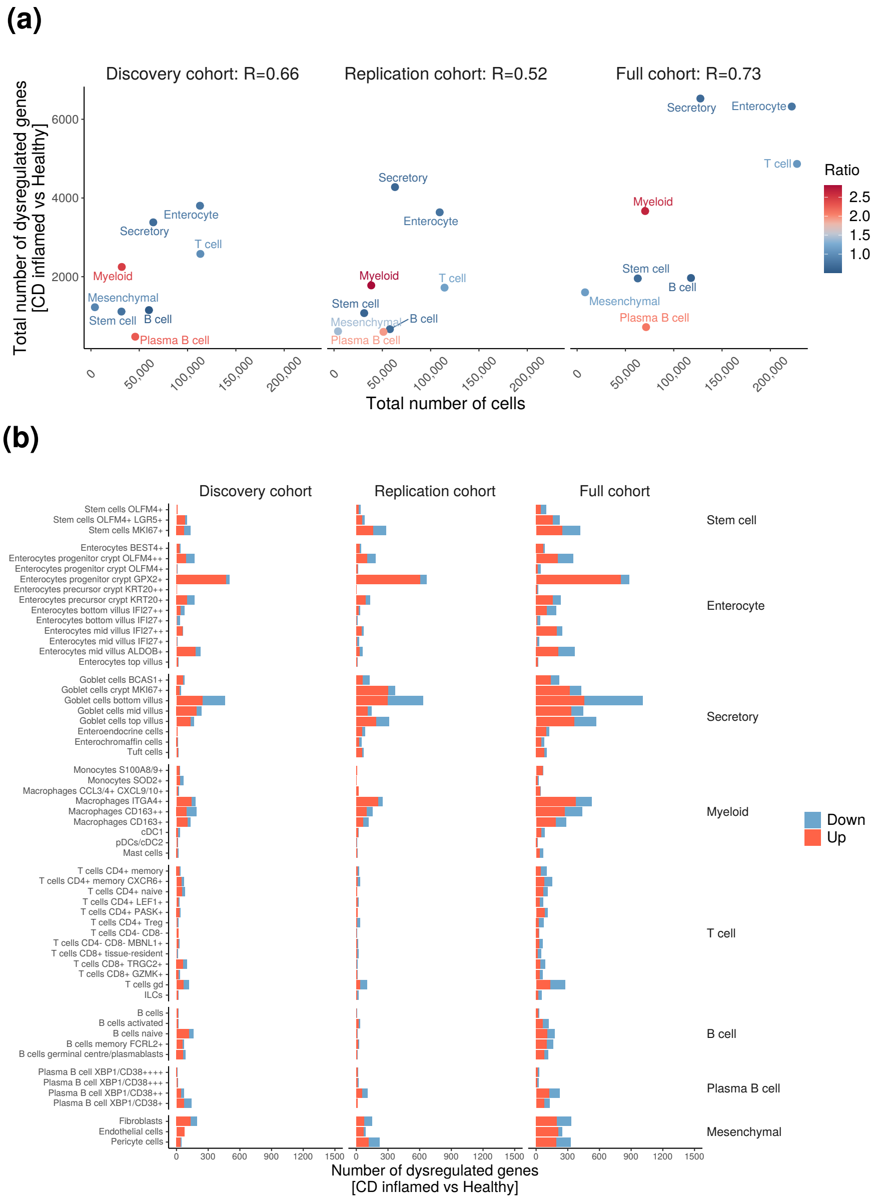

(a) The total number of cells in each major cell population (x-axis), the total number of significantly dysregulated genes (FDR $<$ 5%), and the ratio of cells in Crohn's disease (CD) versus healthy controls (color-coded) across the discovery, replication, and full cohorts. Pearson correlation coefficients (R) were calculated for all major cell populations. **(b)** The number of significantly up- and down-regulated genes (FDR $<$ 5%) is shown on the x-axis for each of the 57 cell types (y-axis) across the discovery, replication, and full cohorts.

Fig. S9. Replicability of differential gene expression results with pseudobulked samples.

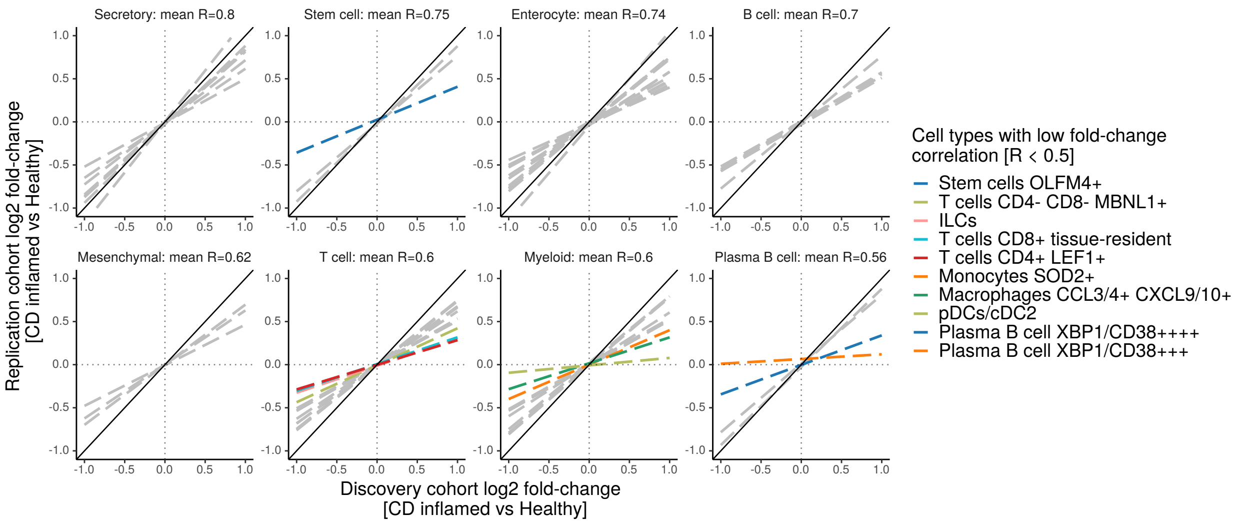

Linear regression (dashed lines) between log2 fold changes of differentially expressed genes (without thresholding, as outlined in Methods) in the pseudobulked discovery (x-axis) and replication (y-axis) datasets. The reported mean Pearson R represents the average of regression coefficients calculated across cell types within each major cell population. Highlighted cell types with low fold-change correlation ($R < 0.5$) were excluded from this analysis.

Fig. S10. Dysregulated pathways in epithelial cells.

(a) MHC-I antigen presentation

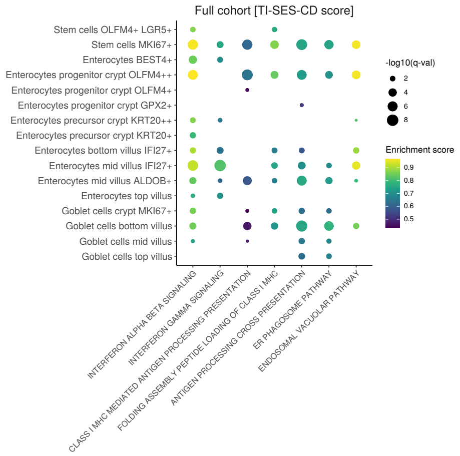

(b) Organoid validation

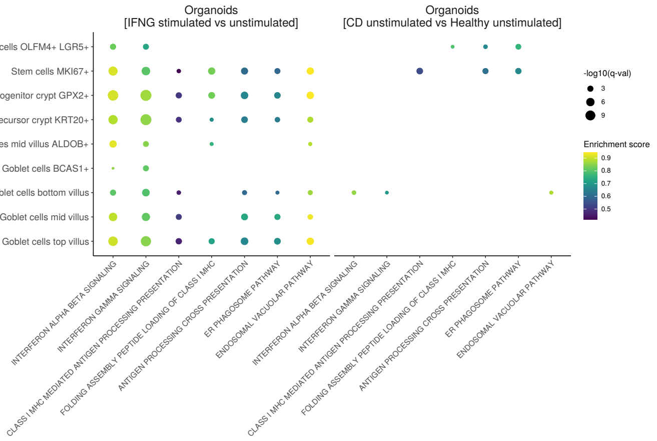

(c) MHC-I vs MHC-II antigen presentation

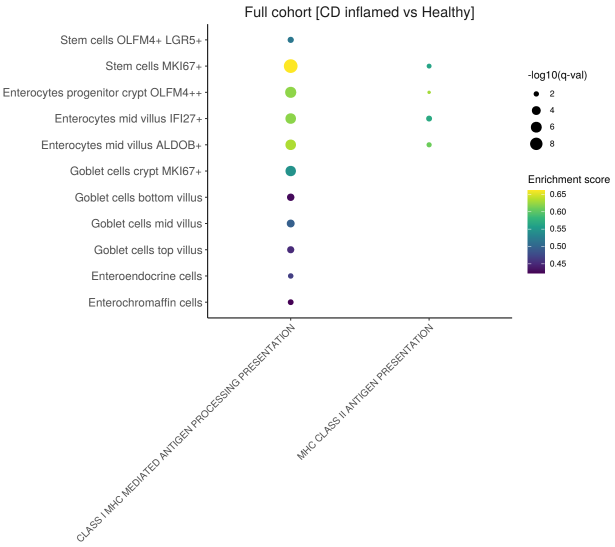

(d) Metabolic pathways

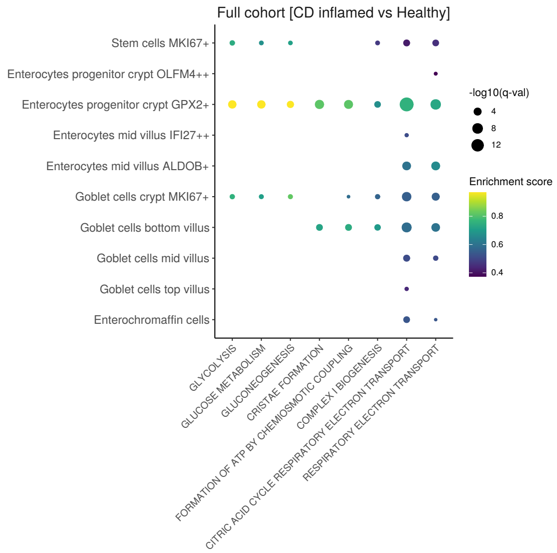

Gene set enrichment analysis results reveal upregulated pathways in epithelial cells, including **(a)** those associated with the TI-SES-CD score, **(b)** organoid-derived cell types both in the presence or absence of interferon gamma as well as between disease and health, **(c)** enrichment of MHC class II antigen presentation compared to MHC class I, and **(d)** significant metabolic changes within epithelial cells. For the terminal ileal biopsy results, analysis was performed on the full cohort with populations exhibiting low fold-change replicability (Pearson's $R < 0.5$, Table S5) excluded.

Uniform Manifold Approximation and Projection (UMAP) of cells from the organoid dataset that met quality control criteria (Methods), with cells coloured by (top) experimental condition (control vs interferon gamma stimulation), (middle) disease status of the donor (healthy vs Crohn's disease), and (bottom) ileal cell type annotation.

Fig. S11. Single-cell expression atlas of ileal-derived organoids stimulated with interferon gamma.

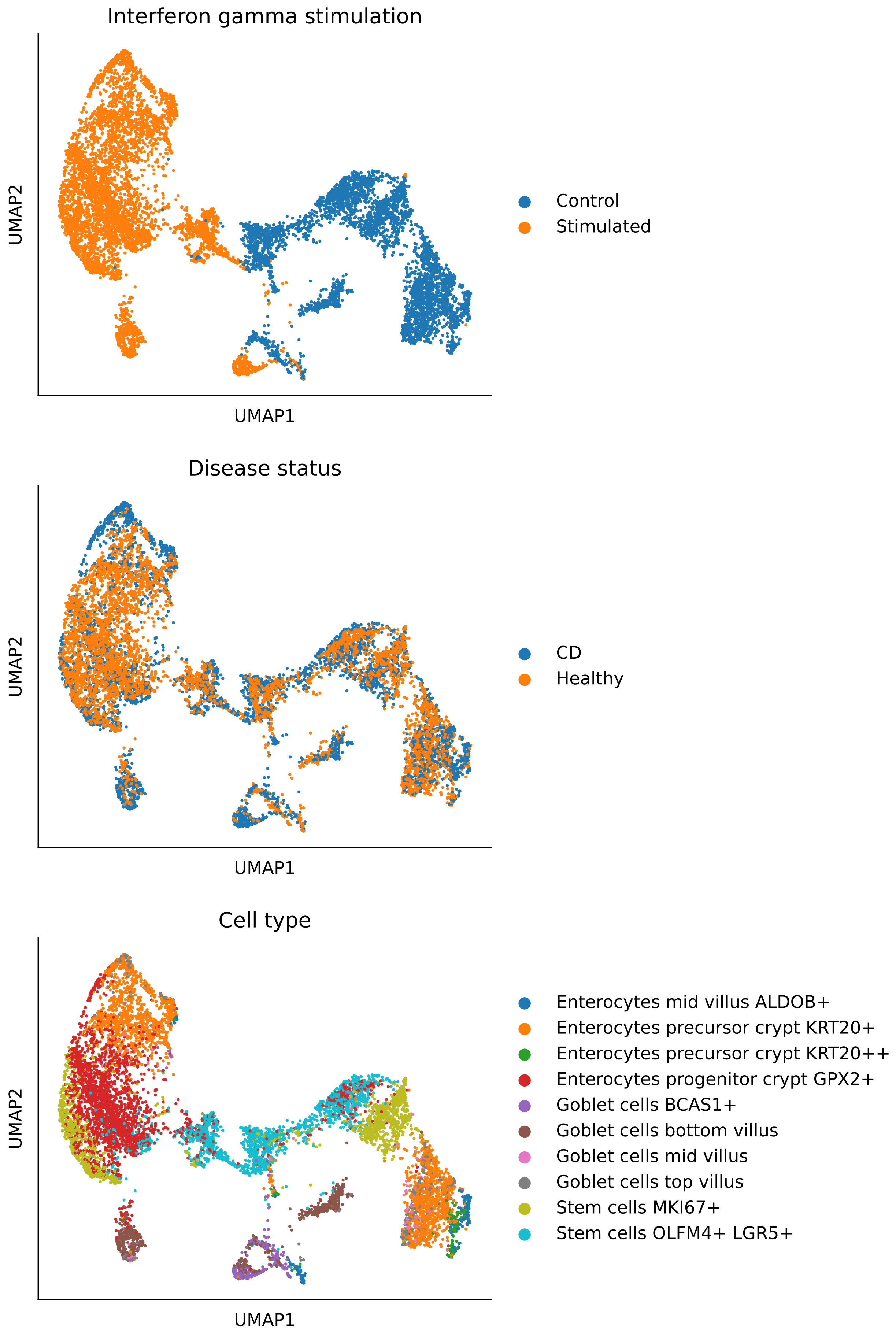

Fig. S12. Myeloid cell types enriched for CD heritability are found predominantly in inflamed CD gut biopsies.

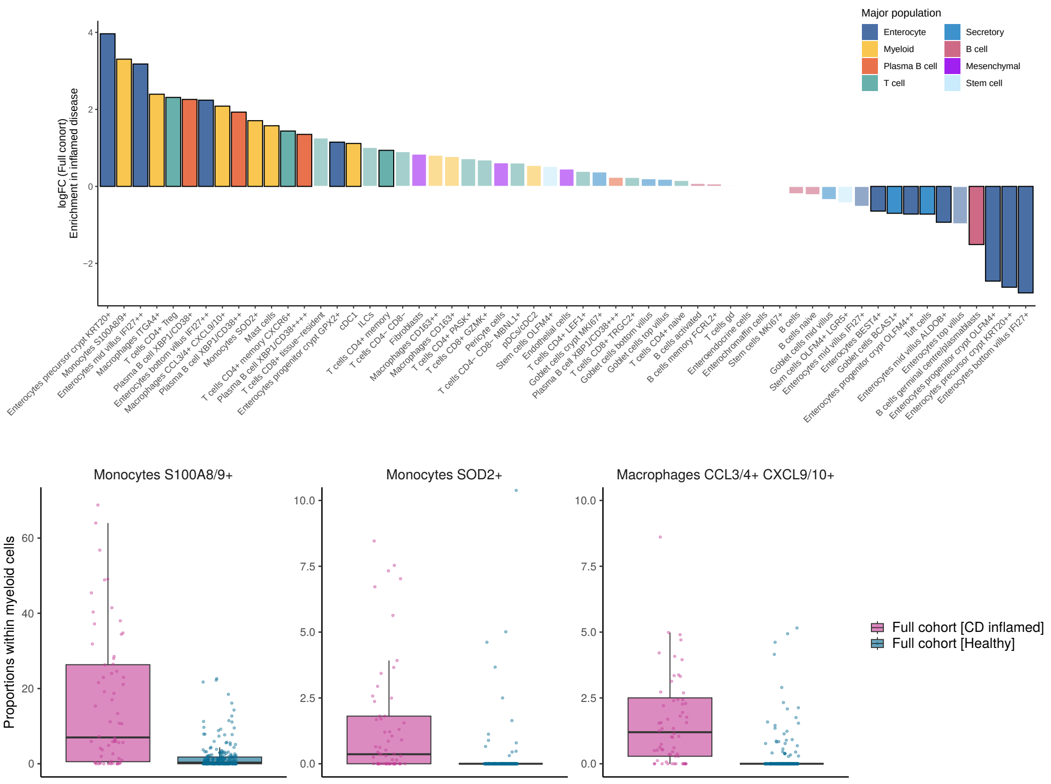

(a) Differential cell-type abundance between health and inflamed Crohn's disease (CD). Values show log fold change (CD [inflamed] vs healthy [uninflamed]); positive values indicate enrichment in CD inflammation and negative values indicate enrichment in healthy controls. Cell types significantly and reproducibly (same direction and FDR < 0.05 in both cohorts) enriched are highlighted and outlined. **(b)** Boxplots show proportions of *S100A8/9+* and *SOD2+* monocytes and *CCL3/4+ CXCL9/10+* macrophages within myeloid cells, comparing inflamed CD and healthy samples. Center line shows the median value, box bounds show the second and third quartile values, and the whiskers show 1.5x the interquartile range.

Fig. S13. Optimisation of cluster resolution for cell-type identification.

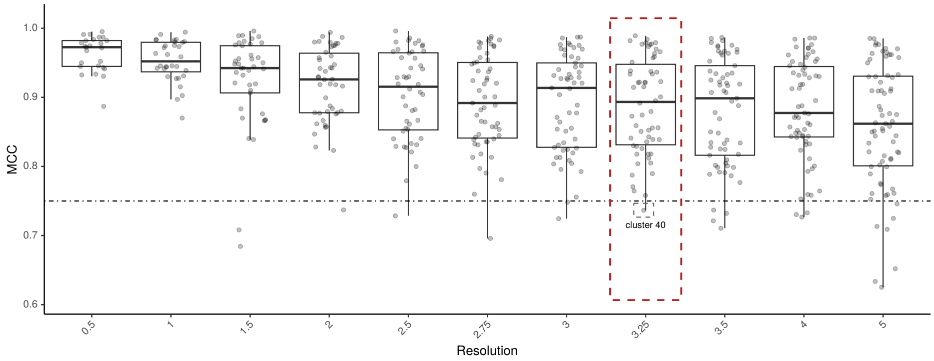

Cluster predictability (x-axis) across a range of clustering resolutions (y-axis). Cluster predictability was assessed by training a keras model on 2/3 of data and calculating the Matthews correlation coefficient (MCC) in the remaining 1/3 test set (Methods). For each boxplot the center line shows the median value, box bounds show the second and third quartile values, and the whiskers show 1.5x the interquartile range. A resolution of 3.25 was selected, as cluster predictability rapidly declined at resolutions greater than 3.25. At this resolution, all clusters met the MCC threshold of > 0.75, with the exception of cluster 40, which exhibited an MCC < 0.75 at multiple resolutions and was therefore excluded.

Supplementary Tables

Table S3. Demographics of samples across cohorts.

	Discovery	Replication	P-value
N	171	172	
Disease status = Healthy (%)	114 (66.7)	118 (68.6)	0.730
Inflammation = Minimal/none (%)	142 (83.0)	137 (79.7)	0.489
TI-SES-CD (%)			0.431
$[0-3)$	142 (83.0)	137 (79.7)	
$[3-6)$	21 (12.3)	29 (16.9)	
$[6-9)$	8 (4.7)	6 (3.5)	
Sex = F (%)	89 (52.0)	88 (51.2)	0.914
Smoking Status (%)			0.640
never	131 (76.6)	124 (72.1)	
yes	22 (12.9)	24 (14.0)	
ex-smoker	15 (8.8)	22 (12.8)	
vape only	3 (1.8)	2 (1.2)	
Disease duration (%)			0.815
Newly diagnosed	4 (2.3)	5 (2.9)	
$[1-10]$ years	23 (13.5)	27 (15.7)	
$[10-20)$ years	15 (8.8)	13 (7.6)	
$[20-30)$ years	9 (5.3)	7 (4.1)	
$[30-40)$ years	4 (2.3)	2 (1.2)	
≥ 40 years	2 (1.2)	0 (0.0)	
Drugs (%)			0.656
Anti-integrin	2 (1.2)	4 (2.3)	
Anti-TNF	10 (5.8)	11 (6.4)	
Azathioprine	9 (5.3)	7 (4.1)	
Azathioprine, Anti-integrin	2 (1.2)	0 (0.0)	
Azathioprine, Anti-TNF	3 (1.8)	3 (1.7)	
None	144 (84.2)	143 (83.1)	
Other	1 (0.6)	4 (2.3)	
TI-SES-CD: ulcers (%)			0.355
0	29 (50.9)	20 (37.0)	
1	18 (31.6)	23 (42.6)	
2	10 (17.5)	10 (18.5)	
3	0 (0.0)	1 (1.9)	
TI-SES-CD: affected surface (%)			0.189
0	28 (49.1)	20 (37.0)	
1	20 (35.1)	29 (53.7)	
2	8 (14.0)	4 (7.4)	
3	1 (1.8)	1 (1.9)	
TI-SES-CD: ulcerated surface (%)			0.229
0	29 (50.9)	20 (37.0)	
1	19 (33.3)	26 (48.1)	
2	9 (15.8)	7 (13.0)	
3	0 (0.0)	1 (1.9)	
Mean age (SD)	46.62 (14.05)	46.49 (12.98)	0.928
Mean genes (SD)	2,401.40 (677.66)	2,405.18 (607.77)	0.957
Mean UMIs (SD)	11,883.43 (4,414.93)	12,111.13 (4,054.32)	0.619

Absolute number and proportions of key variables across discovery and replication cohorts. Non-significant (p-value $\geq$ 0.05) differences determined by two-sided Fisher's exact test (categorical variables) or two-sided t-test (numeric variables). SD=standard deviation.
