# Supplementary material for: Single-cell RNA sequencing of terminal ileal biopsies identifies signatures of Crohn’s disease pathogenesis
Source: Nat Genet. 2026 Jun 15;58(7):1610–9. doi: 10.1038/s41588-026-02634-7 (PMC13364666; doi:10.1038/s41588-026-02634-7)
Supplement: Supplementary file 2 — Reporting Summary [file 41588_2026_2634_MOESM2_ESM.pdf]

nature portfolio

Corresponding author(s): Dr Carl Anderson

Last updated by author(s): Apr 28, 2026

Reporting Summary

Nature Portfolio wishes to improve the reproducibility of the work that we publish. This form provides structure for consistency and transparency in reporting. For further information on Nature Portfolio policies, see our Editorial Policies and the Editorial Policy Checklist.

Statistics

For all statistical analyses, confirm that the following items are present in the figure legend, table legend, main text, or Methods section.

n/a	Confirmed	
☐	☒	The exact sample size (*n*) for each experimental group/condition, given as a discrete number and unit of measurement
☐	☒	A statement on whether measurements were taken from distinct samples or whether the same sample was measured repeatedly
☐	☒	The statistical test(s) used AND whether they are one- or two-sided<br>*Only common tests should be described solely by name; describe more complex techniques in the Methods section.*
☐	☒	A description of all covariates tested
☐	☒	A description of any assumptions or corrections, such as tests of normality and adjustment for multiple comparisons
☐	☒	A full description of the statistical parameters including central tendency (e.g. means) or other basic estimates (e.g. regression coefficient) AND variation (e.g. standard deviation) or associated estimates of uncertainty (e.g. confidence intervals)
☐	☒	For null hypothesis testing, the test statistic (e.g. *F*, *t*, *r*) with confidence intervals, effect sizes, degrees of freedom and *P* value noted<br>*Give P values as exact values whenever suitable.*
☒	☐	For Bayesian analysis, information on the choice of priors and Markov chain Monte Carlo settings
☒	☐	For hierarchical and complex designs, identification of the appropriate level for tests and full reporting of outcomes
☐	☒	Estimates of effect sizes (e.g. Cohen's *d*, Pearson's *r*), indicating how they were calculated

Our web collection on statistics for biologists contains articles on many of the points above.

Software and code

Policy information about availability of computer code

Data collection	CellRanger v7.2.0 was used to demultiplex reads, align reads to GRCh38 with Ensembl version 93 transcript definitions (GRCh38-3.0.0 reference file distributed by 10X Genomics), and generate cell by gene count matrices. CellBender v2.1 [67] was then applied to identify droplets containing cells and adjust the raw counts matrix for background ambient transcript contamination.
Data analysis	List of packages used in data analysis: Python (version 3.7), R (4.0.2), Pandas (version 1.2.3), ScanPy (version 1.9), nextflow (23.04).<br><br>Identify droplets: CellBender v2.1, Estimate of the number of droplets: DropletUtils v1.9.16, Remove multiplets: scrublet v0.2.1, Detect outlier cells: scikit-learn v0.23.2 (isolation forest), Filter, normalise, highly variable genes: scanpy v1.6.0, Calculate optimal number of principal components: kneedle estimator v0.7.0, Calculate principal components: scanpy v1.6.0, Remove batch effects: bbknn v1.3.12, Calculate clusters: Leiden clustering algorithm v0.8.3, Predict cluster identity with single layer dense neural network: keras v2.4.3, Identify gene markers: scanpy v1.6.0, Calculate UMAP projection: scanpy v1.6.0, Auto-annotate cells: Celltypist v1.6.2, Identify differentially expressed genes: MAST v1.14.0, Identify pseudobulk differentially expressed genes: DESeq2 v1.42.1 ,Gene set enrichment analysis: FGSEA v1.17.1, Identify specifically expressed genes: CELLEX v1.2.1, Heritability enrichment analysis: CELLECT v1.3.0, Differential abundance analysis: limma v3.4.2<br><br>The code used for analyses within this study is available at https://github.com/andersonlab/sc_ti_atlas

For manuscripts utilizing custom algorithms or software that are central to the research but not yet described in published literature, software must be made available to editors and reviewers. We strongly encourage code deposition in a community repository (e.g. GitHub). See the Nature Portfolio guidelines for submitting code & software for further information.

Data

Policy information about availability of data

All manuscripts must include a data availability statement. This statement should provide the following information, where applicable:

- Accession codes, unique identifiers, or web links for publicly available datasets
- A description of any restrictions on data availability
- For clinical datasets or third party data, please ensure that the statement adheres to our policy

We have centralized all data exploration, version history, and file access at our project website: https://www.ibdverse.info/. We deposited processed data (single-cell objects, summary statistics, anonymized metadata and models) in the BioStudies database (http://www.ebi.ac.uk/biostudies) under accession numbers S-BSST2944, E-MTAB-16998 and MTAB-16999. Raw sequencing data from the ileal samples and clinical metadata is available in the European Genome-phenome Archive (EGA; https://ega-archive.org) under accession EGAD00001015692, which is accessible following approval by the Sanger Data Access Committee.

Research involving human participants, their data, or biological material

Policy information about studies with human participants or human data. See also policy information about sex, gender (identity/presentation), and sexual orientation and race, ethnicity and racism.

Reporting on sex and gender	Gender was self-reported by all participants through a questionnaire. Gender assignments were subsequently verified by comparing self-reported gender with chromosomal sex data from genotype analysis.
Reporting on race, ethnicity, or other socially relevant groupings	Ethnicity was self-reported by all participants via a questionnaire. Ethnicity assignments were then validated by comparing self-reported data with continental population ancestry, as determined through principal component analysis of genotype data. Individuals of non-European ancestry were excluded from the study.
Population characteristics	A total of 343 terminal ileum samples were collected from healthy controls and Crohn's disease patients, comprising 166 males and 177 females. CD biopsies were classified based on the endoscopic severity of the disease, using the TI-SES-CD score applied to the terminal ileal segment. All participants were of European ancestry and aged between 18 and 70 years. Treatment categories were documented but were not included as covariates in the study.
Recruitment	Individuals undergoing routine endoscopic assessment were recruited at Addenbrooke's hospital, Cambridge, UK. All control participants were undergoing endoscopic assessment or surveillance for healthy and non-cancer related reasons (e.g., history of iron deficiency anaemia, family history of colorectal cancer). Control participants did not have macroscopic evidence of intestinal inflammation, a personal history of cancer, and were not in receipt of corticosteroids or any other immune modulating therapy.
Ethics oversight	This study was approved by the National Health Service (NHS) Research Ethics Committee (Cambridge South, REC ID 17/EE/0338). Written informed consent was given by all participants.

Note that full information on the approval of the study protocol must also be provided in the manuscript.

Field-specific reporting

Please select the one below that is the best fit for your research. If you are not sure, read the appropriate sections before making your selection.

☒ Life sciences ☐ Behavioural & social sciences ☐ Ecological, evolutionary & environmental sciences

For a reference copy of the document with all sections, see nature.com/documents/nr-reporting-summary-flat.pdf

Life sciences study design

All studies must disclose on these points even when the disclosure is negative.

Sample size	Our study analyzed over 1.1 million cells derived from 343 terminal ileal biopsies, including 111 from Crohn's disease patients and 232 from healthy controls, representing the largest scRNA-seq dataset of terminal ileal biopsies to date. In contrast, prior scRNA-seq studies examining differential gene expression in IBD and CD have generally involved smaller cohorts (N < 40) and fewer cells (<430,000). Sample collection in our study was based on donor availability during the designated sampling period, and no statistical methods were used to pre-determine the sample size.
Data exclusions	Patients: To minimize confounding factors, we excluded patients who were using probiotics or antibiotics. Additionally, individuals of non-European ancestry and those outside the age range of 18 to 70 years were not included in the study.<br>Data: Cells were filtered using stringent quality control metrics, which involved the removal of doublets and low-quality cells (see Methods for details).
Replication	A total of 343 biological samples were randomly allocated to either a 'discovery' cohort (57 CD patients and 114 healthy controls) or a 'replication' cohort (54 CD patients and 118 healthy controls). The reproducibility of differential gene expression results varied across different cell types. This variability is likely attributed to the transcriptional plasticity of immune cells, influenced by factors such as diet, inflammation severity, disease stage, and treatment regimens (see Discussion). Organoid data was also generated from 4 CD patients and 3

healthy controls, stimulated to emulate the inflammatory conditions and tested to see if epithelial results replicated.

Randomization: Samples were randomly allocated into two cohorts. No significant differences were observed in baseline demographics between the two cohorts.

Blinding: Samples were anonymized using unique ID numbers prior to data analysis. Investigators used random selection to allocate participants into two independent groups.

Reporting for specific materials, systems and methods

We require information from authors about some types of materials, experimental systems and methods used in many studies. Here, indicate whether each material, system or method listed is relevant to your study. If you are not sure if a list item applies to your research, read the appropriate section before selecting a response.

Materials & experimental systems

n/a	Involved in the study
☒	☐ Antibodies
☒	☐ Eukaryotic cell lines
☒	☐ Palaeontology and archaeology
☒	☐ Animals and other organisms
☒	☐ Clinical data
☒	☐ Dual use research of concern
☒	☐ Plants

Methods

n/a	Involved in the study
☒	☐ ChIP-seq
☒	☐ Flow cytometry
☒	☐ MRI-based neuroimaging

Plants

Seed stocks: *Report on the source of all seed stocks or other plant material used. If applicable, state the seed stock centre and catalogue number. If plant specimens were collected from the field, describe the collection location, date and sampling procedures.*

Novel plant genotypes: *Describe the methods by which all novel plant genotypes were produced. This includes those generated by transgenic approaches, gene editing, chemical/radiation-based mutagenesis and hybridization. For transgenic lines, describe the transformation method, the number of independent lines analyzed and the generation upon which experiments were performed. For gene-edited lines, describe the editor used, the endogenous sequence targeted for editing, the targeting guide RNA sequence (if applicable) and how the editor was applied.*

Authentication: *Describe any authentication procedures for each seed stock used or novel genotype generated. Describe any experiments used to assess the effect of a mutation and, where applicable, how potential secondary effects (e.g. second site T-DNA insertions, mosiacism, off-target gene editing) were examined.*
