# Supplementary material for: Single-cell RNA sequencing of terminal ileal biopsies identifies signatures of Crohn’s disease pathogenesis
Source: Nat Genet. 2026 Jun 15;58(7):1610–9. doi: 10.1038/s41588-026-02634-7 (PMC13364666; doi:10.1038/s41588-026-02634-7)
Supplement: Supplementary file 3 — Peer Review File [file 41588_2026_2634_MOESM3_ESM.pdf]

nature portfolio

Peer Review File

Single-Cell RNA Sequencing of Terminal Ileal Biopsies Identifies Signatures of Crohn’s Disease Pathogenesis.

Corresponding Author: Dr Carl Anderson

This file contains all editorial decision letters in order by version, followed by all author rebuttals in order by version.

Version 0:

Decision Letter:

6th Mar 2025

Dear Dr Anderson,

First, please accept my apologies for the delay in returning this decision to you. Thank you for bearing with me.

Your Analysis, "Single-Cell RNA Sequencing of Terminal Ileal Biopsies Identifies Signatures of Crohn’s Disease Pathogenesis." has now been seen by 2 referees. You will see from their comments copied below that while they find your work of considerable potential interest, they have raised quite substantial concerns that must be addressed. In light of these comments, we cannot accept the manuscript for publication, but would be very interested in considering a revised version that addresses these serious concerns.

We hope you will find the referees' comments useful as you decide how to proceed. If you wish to submit a substantially revised manuscript, please bear in mind that we will be reluctant to approach the referees again in the absence of major revisions. Please bear in mind that we might also need to send the paper out to a new reviewer(s), although we will only do this if necessary.

If you choose to revise your manuscript taking into account all reviewer and editor comments, please highlight all changes in the manuscript text file. At this stage we will need you to upload a copy of the manuscript in MS Word .docx or similar editable format.

We are committed to providing a fair and constructive peer-review process. Do not hesitate to contact us if there are specific requests from the reviewers that you believe are technically impossible or unlikely to yield a meaningful outcome.

If revising your manuscript:

*1) Include a “Response to referees” document detailing, point-by-point, how you addressed each referee comment. If no action was taken to address a point, you must provide a compelling argument. This response will be sent back to the referees along with the revised manuscript.

*2) If you have not done so already please begin to revise your manuscript so that it conforms to our Analysis format instructions, available here.
Refer also to any guidelines provided in this letter.

*3) Include a revised version of any required Reporting Summary: https://www.nature.com/documents/nr-reporting-summary.pdf
It will be available to referees (and, potentially, statisticians) to aid in their evaluation if the manuscript goes back for peer review.
A revised checklist is essential for re-review of the paper.

Please be aware of our guidelines on digital image standards.

EXTENDED DATA FIGURES

When re-submitting your manuscript, please ensure that any supplementary figures and tables that are crucial to the manuscript's conclusions are converted into Extended Data figures and tables to increase visibility of these data. Extended Data figures and tables are online-only (present in the online PDF and full-text HTML versions of the paper), peer-reviewed display items that provide essential background to the article but are not included in the main article due to space constraints. A maximum of ten Extended Data display items (figures and tables) is permitted.

You may use the link below to submit your revised manuscript and related files:

Link Redacted

Note: This URL links to your confidential home page and associated information about manuscripts you may have submitted, or that you are reviewing for us. If you wish to forward this email to co-authors, please delete the link to your homepage.

If you wish to submit a suitably revised manuscript we would hope to receive it within 6 months. If you cannot send it within this time, please let us know. We will be happy to consider your revision so long as nothing similar has been accepted for publication at Nature Genetics or published elsewhere. Should your manuscript be substantially delayed without notifying us in advance and your article is eventually published, the received date would be that of the revised, not the original, version.

Please do not hesitate to contact me if you have any questions or would like to discuss the required revisions further.

Nature Genetics is committed to improving transparency in authorship. As part of our efforts in this direction, we are now requesting that all authors identified as 'corresponding author' on published papers create and link their Open Researcher and Contributor Identifier (ORCID) with their account on the Manuscript Tracking System (MTS), prior to acceptance. ORCID helps the scientific community achieve unambiguous attribution of all scholarly contributions. You can create and link your ORCID from the home page of the MTS by clicking on 'Modify my Springer Nature account'. For more information please visit please visit www.springernature.com/orcid.

Thank you for the opportunity to review your work.

Sincerely,

Safia Danovi, PhD
Senior Editor, Nature Genetics
ORCiD: 0009-0007-7822-5479

Referee expertise:

Referee #1: sc, Crohn's disease

Referee #2: sc

Reviewers' Comments:

Reviewer #1 (Remarks to the Author):

I read with interest Krzak et al's manuscript entitled "Single-Cell RNA Sequencing of Terminal Ileal Biopsies Identifies Signatures of Crohn's Disease Pathogenesis." The authors report what seems to be the largest scRNA-seq dataset of terminal ileal biopsies, derived from CD patients and controls. This in and of itself is an achievement, and this atlas will likely serve as a robust resource for future investigation. The focus of the manuscript is primarily related to atlassing, quality control metrics, and a handful of key findings all based on in silico data. The authors main findings related to signatures of inflammation include interferon and MHC responses in inflamed epithelial cells, and enriched cytokine signaling in ITGA4+ macrophages. These findings in and of themselves are not entirely novel (although perhaps these signals are shown most robustly in their powerful dataset), but rather corroborate aspects of what is already known in the IBD literature. Ultimately, the presentation of analysis and data is limited by some analytic concerns detailed below as well as the lack of mechanistic (or even any form of validation experiment such as IHC etc) exploration of the main claims.

Major edits/points of improvement:
1) The authors seem to use individual as a random effect in their model (in an effort to overcome pseudoreplication bias as per Zimmerman et al, Nature Communications 2021). However, since then there has been work suggesting flaws in this approach and that pseudobulk methods are superior when conducting differential expression analysis in scRNA-seq data (see Murphy and Skene, Nature Communications, 2022). I wonder whether the authors' observed discrepancies in replicability between their discovery and replication cohorts could relate to their chosen methodology, or how their QC

metrics related to this might change (or improve?) if using a pseudobulk approach. I would suggest using an alternative approach for their DEG analysis, perhaps in a subset of the data, to see if their QC metrics would change for the better.
2) The authors do not mention or include key variables in clinical metadata that might affect analysis, such as treatment/medications, and also important clinical features of CD such as stricturing disease or fistulae. This information should be included as part of clinical metadata but also should be incorporated in the narrative.
3) The authors discuss epithelial interferon signatures at length – but do not describe whether this was unique to epithelial cells or perhaps could be found in other compartments like stromal cells or subsets of lymphocytes.
4) Despite atlassing, the authors do not describe any findings in stromal cells or in the majority of lymphocytes. It is unclear why the focus ended up being just on epithelial cells and ITGA4+ macrophages. If there were no significant findings in the other populations this should be reported as it would go against what others have found in smaller cohorts.
5) Would recommend in vitro work, at minimum, to validate some of key findings.

Reviewer #2 (Remarks to the Author):

In this manuscript, the authors present IBDverse, a large single-cell RNA atlas of terminal ileal biopsies, comprising 1.1 million cells from 111 Crohn's disease (CD) patients and 232 healthy controls. They utilize the data to identify differentially expressed genes, pathways, and cell types associated with CD. The dataset is a valuable addition to the single-cell community, particularly for analyses of cell state dynamics in intestinal disease. However, given the scale of the dataset, the analytical depth appears relatively superficial and should be enhanced in the revised manuscript.

1. Cell Population Differences: The authors report cell population changes between CD and control samples (lines 139–146). Are these differences statistically significant? The CD samples show an enriched population of progenitor cells. Could this be influenced by the relatively younger age of the CD cohort?

2. Quality Control Metrics: Critical quality control statistics are missing, including the number of UMIs/genes detected per cell and the evaluation of batch effect across different experiment/sequencing batches. Integration with published datasets to validate the annotated cell types would further strengthen the study.

3. Differential Expression Analysis: The low consistency of DE genes between the discovery and replication cohorts should be further investigated. Could this be due to the analysis pipeline (e.g., potential high false discovery rates in single-cell DE analysis, see https://www.nature.com/articles/s41467-021-25960-2)? Does the low consistency correlate with the number of cells per sample?

4. Pathway and Cell Linkage Analyses: Beyond DE genes, is the pathway analysis and cell linkage analysis consistent between the discovery and replication cohorts?

5. Gender Distribution: What is the male and female distribution within the samples? Have any gender-specific differences been identified?

6. Transcription Factor Analysis: In addition to DE gene analysis, it is suggested that the authors perform TF–gene regulatory network analysis to identify TF regulators underlying cell state changes in CD samples.

7. The labels for Figure 3a/b are misassigned as Figure S3a/b in the manuscript.

8. The IBDverse website is currently not functional. This should be fixed before the manuscript is published.

Reviewer #2 (Remarks on code availability):

The provided code repository (https://github.com/andersonlab/sc_ti_atlas) appears to be linked to the computational analyses in Krzak et al. 2023 rather than to this study. These issues should be addressed to ensure reproducibility and transparency.

Reviewer #2 (Remarks on figshare data availability):

The data are currently not available via Zenodo or the IBDverse website as listed in the manuscript.

Version 1:

Decision Letter:

Our ref: NG-AN67427R

6th Oct 2025

Dear Carl,

I hope you're well.

Thank you for submitting your revised manuscript "Single-Cell RNA Sequencing of Terminal Ileal Biopsies Identifies Signatures of Crohn’s Disease Pathogenesis." (NG-AN67427R). It has now been seen by the original referees and their comments are below. The reviewers find that the paper has improved in revision, and therefore we'll be happy in principle to publish it in Nature Genetics, pending minor revisions to satisfy our editorial and formatting guidelines.

If the current version of your manuscript is in a PDF format, please email us a copy of the file in an editable format (Microsoft Word or LaTex)-- we can not proceed with PDFs at this stage.

We are now performing detailed checks on your paper and will send you a checklist detailing our editorial and formatting requirements soon. Please do not upload the final materials and make any revisions until you receive this additional information from us.

Thank you again for your interest in Nature Genetics Please do not hesitate to contact me if you have any questions.

Sincerely,

Safia Danovi, PhD
Senior Editor, Nature Genetics
ORCiD: 0009-0007-7822-5479

Reviewer #1 (Remarks to the Author):

I have reviewed the revised version of Krzak et al’s manuscript and the authors’ detailed responses to my prior comments. I am satisfied that they have adequately addressed the concerns I raised (important improvements summarized below):
1. They performed a pseudobulk re-analysis in response to my suggestion and showed that the findings are consistent with their original approach, strengthening confidence in their results.
2. They have expanded and clarified the clinical metadata, including treatment and disease phenotype information – even though they could not analyze these subcategories per se in the manuscript, the data is now available and their rationale is understood.
3. They have clarified the breadth and specificity of the epithelial interferon signature; Importantly, they performed additional in vitro validation using intestinal organoids, which directly supports and strengthens their key findings regarding IFN-driven MHC-I signaling in epithelial cells.
In light of these revisions, I believe the authors have addressed my concerns appropriately.

Reviewer #2 (Remarks to the Author):

The authors have satisfactorily resolved the majority of my concerns. Additional TF–gene linkage analysis could strengthen the work by identifying key TF regulators underlying disease-associated cellular dynamics. Although the authors do not address this comment, I do not consider it necessary for publication.

Reviewer #2 (Remarks on code availability):

The updated website fixes the previous problem.

Open Access This Peer Review File is licensed under a Creative Commons Attribution 4.0 International License, which permits use, sharing, adaptation, distribution and reproduction in any medium or format, as long as you give appropriate credit to the original author(s) and the source, provide a link to the Creative Commons license, and indicate if changes were made.
In cases where reviewers are anonymous, credit should be given to 'Anonymous Referee' and the source.
The images or other third party material in this Peer Review File are included in the article's Creative Commons license, unless indicated otherwise in a credit line to the material. If material is not included in the article's Creative Commons license and your intended use is not permitted by statutory regulation or exceeds the permitted use, you will need to obtain permission directly from the copyright holder.
To view a copy of this license, visit https://creativecommons.org/licenses/by/4.0/

Reviewer #1 (Remarks to the Author):

Major edits/points of improvement:
1) The authors seem to use individual as a random effect in their model (in an effort to overcome pseudoreplication bias as per Zimmerman et al, Nature Communications 2021). However, since then there has been work suggesting flaws in this approach and that pseudobulk methods are superior when conducting differential expression analysis in scRNA-seq data (see Murphy and Skene, Nature Communications, 2022). I wonder whether the authors' observed discrepancies in replicability between their discovery and replication cohorts could relate to their chosen methodology, or how their QC metrics related to this might change (or improve?) if using a pseudobulk approach. I would suggest using an alternative approach for their DEG analysis, perhaps in a subset of the data, to see if their QC metrics would change for the better.

We agree with the reviewer that the single-cell field has yet to conclusively determine whether a single-cell mixed-effects or pseudobulk fixed-effects approach is better suited for differential gene expression (DE) analysis. While pseudobulk methods are increasingly favoured for their robustness in modelling biological replication (e.g. Murphy and Skene, 2022), single-cell mixed models, such as those described by Zimmerman et al. (2021), remain widely used, particularly when cell-level resolution is of interest. Our original analysis used a mixed-effects model with individual as a random effect to account for inter-individual variability while leveraging cell-level information.

In response to the reviewer's suggestion, we re-analysed the full dataset using a pseudobulk DGE approach. The findings were highly consistent with those from our original analysis, including the sets of DE genes and their effect sizes. Unfortunately, the replication rates between the discovery and replication cohorts did not improve, suggesting that differences in replicability are unlikely to be driven by the DE methodology. For these reasons, we have retained the mixed-effects model as our primary analysis. To clarify for readers that the observed replication rates are not driven by the choice of DE methodology, we have included the replication rates from the pseudobulk analysis as a new supplementary figure (see below) and added the following statement to the main text highlighting the consistency of results across approaches:

"We note that these patterns were not substantially altered when using a pseudobulk differential expression method, suggesting that replication rates were not overly influenced by the choice of DGE approach (Fig. S9)."

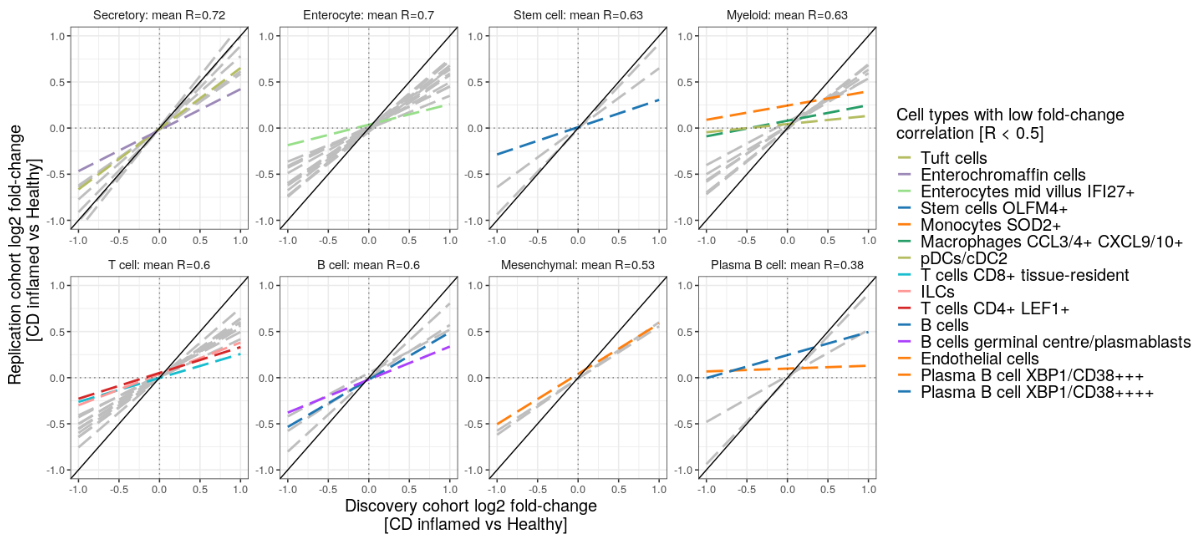

2) The authors do not mention or include key variables in clinical metadata that might affect analysis, such as treatment/medications, and also important clinical features of CD such as stricturing disease or fistulae. This information should be included as part of clinical metadata but also should be incorporated in the narrative.

We agree with the reviewer that clinical metadata, including treatment and key Crohn's disease features, are important contextual variables. In designing our study, we carefully considered - and in some cases agonised over - which clinical subphenotypes or traits to include in differential expression (DE) analyses. Given our central focus on replicability and robustness, we deliberately chose not to pursue DE analyses for phenotypes where sample sizes were too small in either the discovery or replication cohorts to support well-powered, interpretable results.

In response to the reviewer's suggestion, we have now collated additional metadata on treatment and disease phenotype, including duration of disease, treatment class and the subcategories of the SES-CD score. We have incorporated additional disease data into the patient metadata. Because some of this data can be personally identifiable we have shifted this data from supplementary table 1 to EGA where access can be controlled. We hope this will enable other researchers to explore these clinical dimensions in more detail, perhaps via meta-analyses to increase power to detect differentially expressed genes.

3) The authors discuss epithelial interferon signatures at length – but do not describe whether this was unique to epithelial cells or perhaps could be found in other compartments like stromal cells or subsets of lymphocytes.

Type I IFN receptors (IFNAR1/2) are expressed by all nucleated cells and can sense all 17 subtypes of type I IFN. However, Type I IFN responses are often cell type and sub-population specific, with even homogenous populations of cells demonstrating subsets that become refractory to Type I IFN stimulation *in vitro*, with negative feedback to limit IFN response a hallmark of IECs in health (PMID: 38518661, PMID: 33362795).

Our work builds on these previous findings by demonstrating that IEC response to type I IFN in IBD is agnostic to differentiation state, because - in contrast to *in vitro* data - the type I IFN pathway is enriched across almost all epithelial subtypes in our data.

While we do observe significant upregulation of these pathways in many cell types (192 enrichments across our six pathways in both CD inflamed vs healthy and CD uninflamed vs healthy), the majority of these were found in epithelial cell types and 47 of the top 50 enrichments (by normalised enrichment score) were found in epithelial cell types.

Importantly, a key consequence of type I IFN stimulation, MHC-I signalling, remains persistently active selectively in IECs after inflammation has resolved, which we hypothesise may facilitate cross-presentation of luminal antigen in the post-repair state.

We have incorporated these points into the revised manuscript results:

"Whilst we did observe significant upregulation of these pathways across many cell types (192 enrichments across six pathways in both CD inflamed vs healthy and CD uninflamed vs healthy), the epithelium was over-represented, with 47 of the top 50 enrichments (by normalised enrichment score) and 101 out of the 192 enrichments found in epithelial cell types."

4) **Despite atlassing, the authors do not describe any findings in stromal cells or in the majority of lymphocytes. It is unclear why the focus ended up being just on epithelial cells and ITGA4+ macrophages. If there were no significant findings in the other populations this should be reported as it would go against what others have found in smaller cohorts.**

We thank the reviewer for this comment. We agree that stromal and lymphocyte populations have been implicated in prior single-cell studies of Crohn's disease, and we carefully considered whether to highlight findings in

these compartments. However, a central tenet of our study is the assessment of replicability across two cohorts. We therefore chose to focus on epithelial cells and ITGA4+ macrophages, as these were among the cell types that showed the most robust and consistent differential expression in both the discovery and replication cohorts. In contrast, many lymphocyte populations — including the majority of T and B cell subsets — demonstrated poor fold-change replicability, and we did not feel it was appropriate to highlight significant findings from these cell types in the main text. Stromal cells were also relatively underrepresented in our dataset, likely due to the use of small pinch biopsies and a dissociation protocol optimised for epithelial cell recovery. This limited our power to detect differential expression in stromal populations and likely contributed to the observed lack of replicability.

While previous single-cell studies of Crohn's disease have tended to focus more heavily on immune and stromal compartments, our findings place a stronger emphasis on epithelial cell dynamics. We see this as a strength and a novel contribution, enabled by the scale and design of our study and the robust replication of epithelial signals across cohorts. To support transparency and maximise the utility of our dataset, results from all analysed cell types — including lymphocyte and stromal populations not discussed in the main text — will be made available as part of the IBDverse data resource released alongside this manuscript.

5) Would recommend in vitro work, at minimum, to validate some of key findings.

We appreciate the recommendation from the reviewer and agree that *in vitro* validation experiments can support the key findings from our work. Given that IFN driven MHC-1 gene upregulation in IECs was one of these key findings, we elected to obtain primary human intestinal tissue from CD and healthy controls, to generate patient-derived IEC organoids for downstream analysis.

Here, we differentiated CD or healthy IECs from 4 and 3 individuals, respectively, in the presence or absence of IFN (we chose IFNγ, to replicate the inflamed environment). We then performed scRNA-seq to understand if subset-specific gene expression responses to IFN existed in *ex vivo* stimulated cells. First, we were able to successfully align the annotation of IEC subsets from our atlassing dataset to our patient-derived organoids, demonstrating the robustness of our annotation. Second, and in keeping with our presented work, IFN stimulation conferred upregulation of MHC-1 gene networks in pathway analysis. Third, and also in keeping with our initial analyses, pathway enrichment was not confined to an IEC subtype, with a consistent response agnostic to IEC differentiation state.

Remarkably, despite a period of culture *ex vivo* removed from the CD microenvironment, we were still able to detect differences in MHC-1 signalling and cross-presentation between unstimulated CD and healthy control IECs; notably with pathway enrichment within intestinal stem cells from CD patients. This is in keeping with our original findings, where we show that IECs retain some of the features of increased IFN-MHC signalling despite inflammation resolution.

Taken together, we are now able to validate and confirm our initial findings in primary human intestinal tissue from CD patients and healthy controls, demonstrating directly that IFN stimulation is sufficient for enrichment of MHC-1 signalling across IEC subtypes. Furthermore, we show that despite baseline upregulation of MHC-1 signalling in CD ISCs, CD IECs do not demonstrate IFN desensitisation, with non-inferior IFN responses to healthy controls, suggesting that tonic low-level IFN stimulation may exist in the post-inflammatory CD mucosa.

We have added the two figures below as supplementary figure S12 and panel S11b, respectively. We have also added the following paragraph to the results section (The Ileum undergoes both pan-epithelial and cell-type specific changes in CD inflammation):

"*To validate our findings and address the possibility of confounding present in biopsy data (e.g., medication exposure), we generated intestinal organoid scRNA-seq using ileal biopsies from three healthy and four CD individuals. Organoids were either left unstimulated or treated with interferon-γ (IFNγ) to emulate the inflamed intestinal state (Methods). Twelve epithelial cell types were annotated with high confidence in both stimulated and unstimulated organoids (Fig. S12), enabling paired comparisons of stimulation-induced transcriptional changes. Consistent with our primary tissue analyses, IFNγ stimulation induced upregulation of "IFNγ signalling" alongside MHC-I antigen-processing pathways, including "Folding-assembly, peptide loading of class I MHC", "Antigen processing: cross-presentation", "ER phagosome", and "Endosomal vacuolar pathway", across the majority of epithelial cell types (Fig. S11b), supporting an epithelial-intrinsic antigen-presentation response in inflammation. We also performed a comparison between health and disease in the unstimulated organoids and, in line with the biopsy data, we observed a weak enrichment of selected MHC-I pathways (Fig. S11b), indicating that some elements of the antigen-presentation programme persist outside the inflamed micro-environment.*"

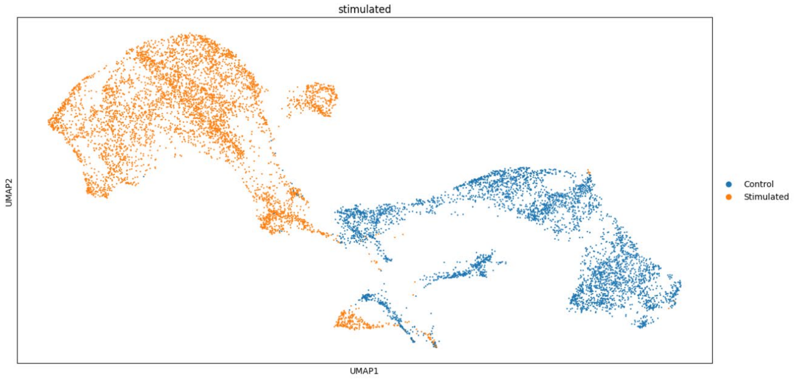
stimulated
UMAP2
UMAP1
Control
Stimulated

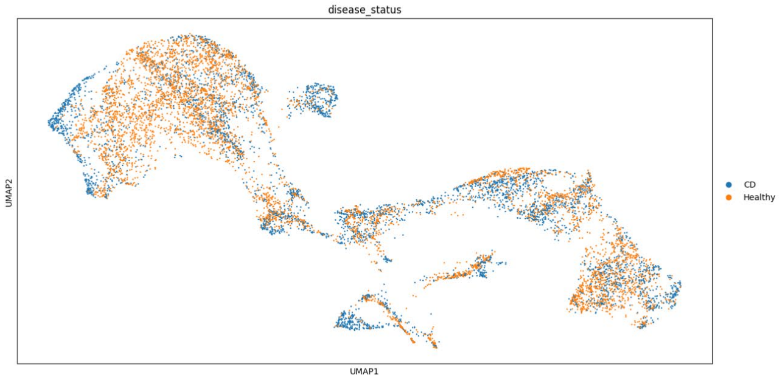
disease_status
UMAP2
UMAP1
CD
Healthy

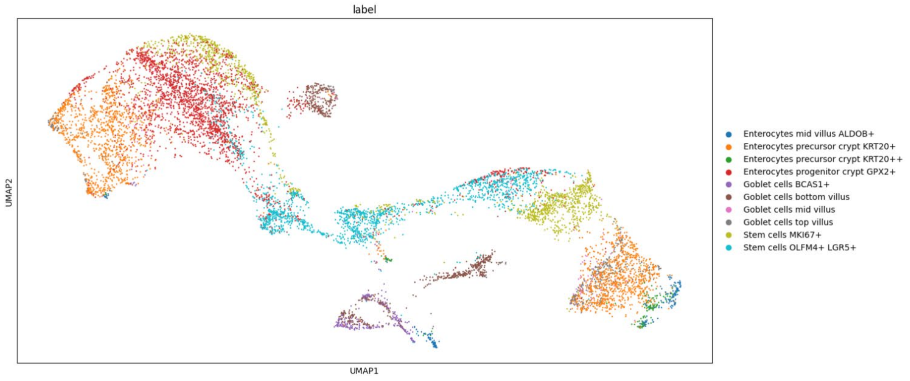
label
UMAP2
UMAP1
Enterocytes mid villus ALDOB+
Enterocytes precursor crypt KRT20+
Enterocytes precursor crypt KRT20++
Enterocytes progenitor crypt GPX2+
Goblet cells BCAS1+
Goblet cells bottom villus
Goblet cells mid villus
Goblet cells top villus
Stem cells MKI67+
Stem cells OLFM4+ LGR5+

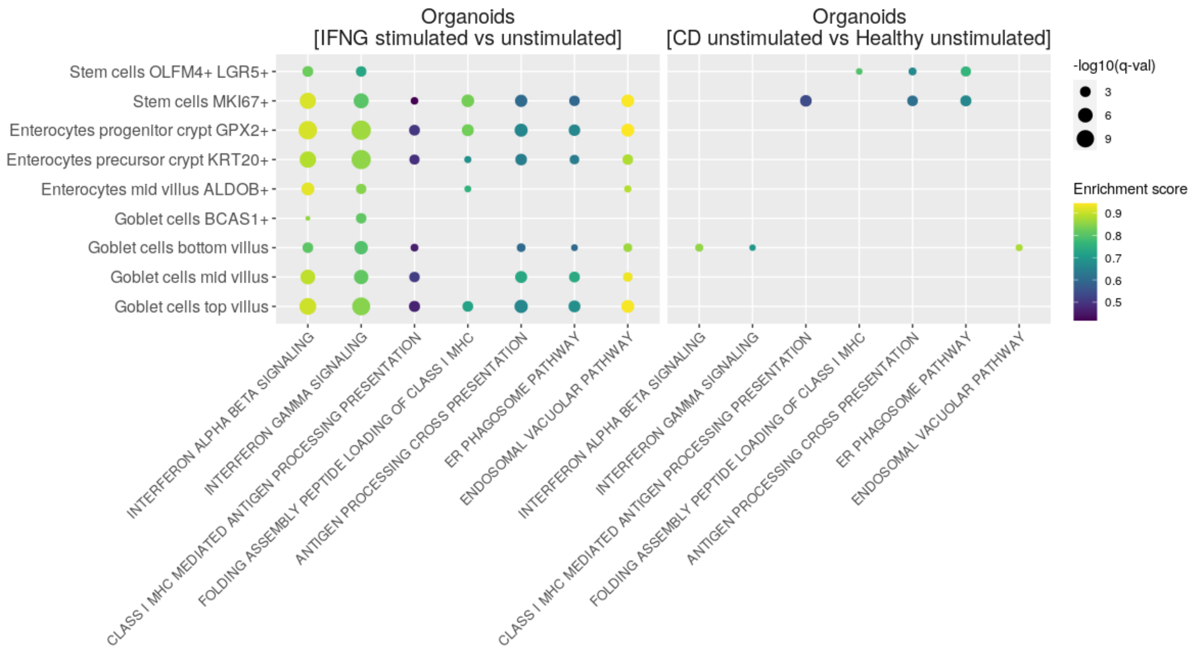

Reviewer #2 (Remarks to the Author):

1. Cell Population Differences: The authors report cell population changes between CD and control samples (lines 139–146). Are these differences statistically significant? The CD samples show an enriched population of progenitor cells. Could this be influenced by the relatively younger age of the CD cohort?

We thank the reviewer for this comment. We initially chose not to undertake comprehensive statistical tests for differences in cell type proportions between Crohn's disease (CD) cases and controls because, in the early stages of the study, we intentionally adjusted the representation of cell types, particularly in CD samples, to ensure adequate epithelial cell recovery in the context of the substantial immune cell infiltration characteristic of inflamed tissue. After applying this approach to approximately 70 samples, we found that such manipulation was unnecessary, and the remaining 270 samples were processed without intervention.

In response to the reviewer's suggestion, we have formally tested for differences in cell type abundance between cases and controls, here cases are all CDs and all inflamed CDs. For each comparison we fit a differential abundance model (~disease_status + age_binned + sex + fractioned) within all three cohorts, with a simple binary variable "fractioned" indicating whether the sample had been adjusted or not. Thirteen of 57 cell types were significantly positively associated with disease (FDR < 0.05, log fold change > 0) in the discovery, replication and full cohorts. These included the two myeloid populations highlighted in the manuscript, S100A8/9+ monocytes and

CXCL9/CXCL10+ macrophages, as well as the four enterocyte populations which we report - GPX2+ progenitor crypt, KRT20+ precursor crypt, IFI27++ mid-villus, and IFI27++ bottom-villus enterocytes - which were the only epithelial cell types significantly enriched in disease. In the inflamed CD comparison we see greater log fold changes and several more cell types that are significantly associated, such as cDC1s. The inclusion of age as a covariate in the model indicates that these findings are unlikely to be explained by the relatively younger age of the CD cohort.

We have added the figures shown below as additional panels in Figure 1 and in Figure S13. We have also added the following to the main text:

"*Statistical testing identified 13 of 57 cell types as significantly enriched in CD in both cohorts (false discovery rate [FDR] < 5%, log fold change > 0), including several known to accumulate in intestinal inflammation (Figure 1C)*".

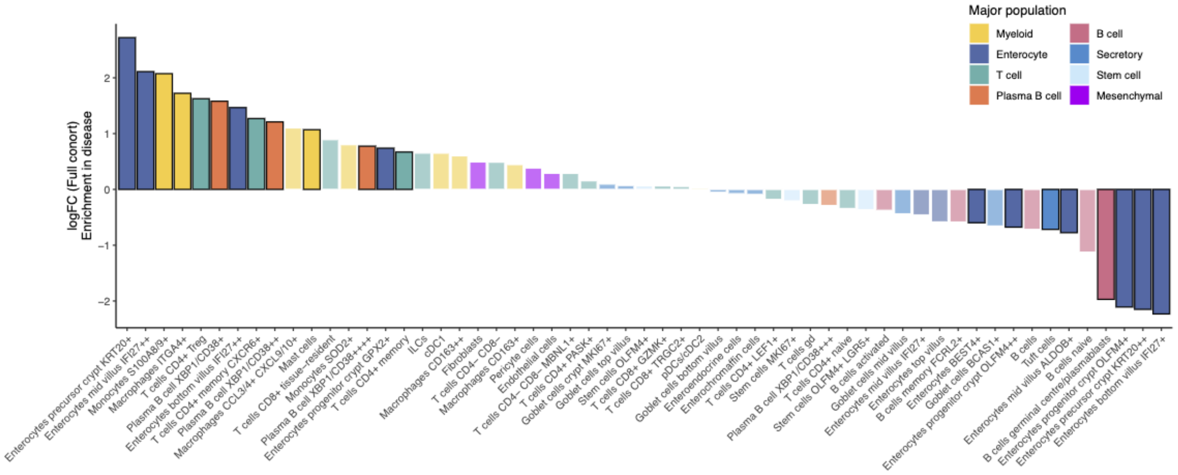

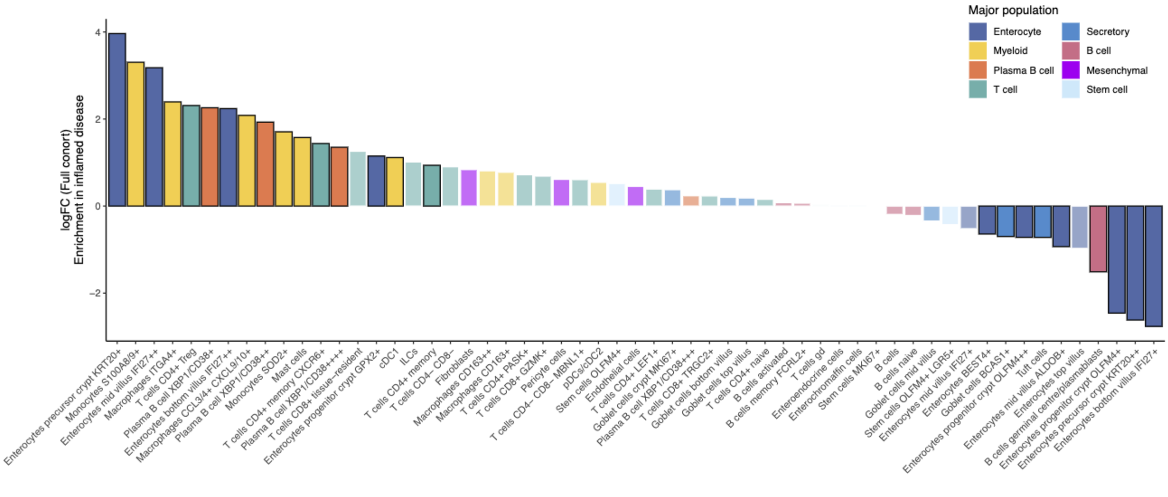

2. Quality Control Metrics: Critical quality control statistics are missing, including the number of UMIs/genes detected per cell and the evaluation of batch effect across different experiment/sequencing batches. Integration with published datasets to validate the annotated cell types would further strengthen the study.

We thank the reviewer for alerting us to the lack of quality control data and agree that these are critical to interpret the presented results. We have added additional plots characterising the distribution of key quality control metrics (number of UMIs, number of genes and mitochondrial percentage) across disease and the 8 major populations which we capture in our data, in both the atlasing cohort (a-c) and full cohort (d-f). We purposefully relaxed some of our atlasing cohort QC thresholds, when applied to the full cohort. This enabled us to capture the greatest biological diversity while simultaneously mapping cells confidently using our cell type classifier onto a robust annotation atlas.

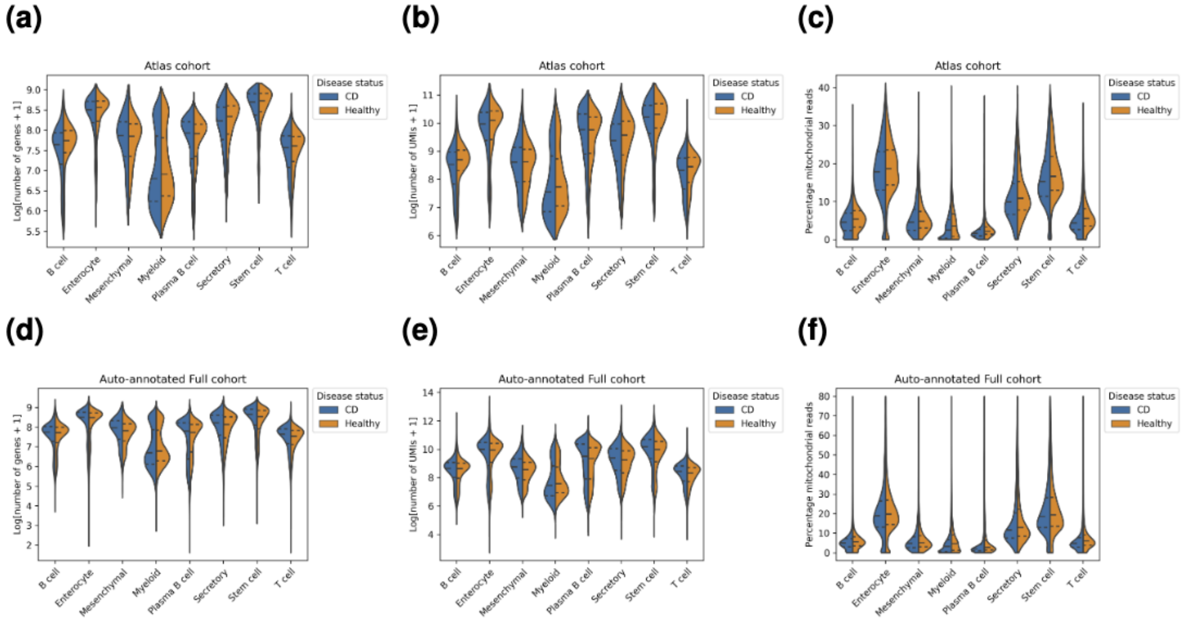

Following the reviewer's suggestion, we now show evidence of successful integration across batches via UMAPs coloured by the potential technical covariates that might cause batch effects in our data. When performing batch correction, we corrected over the sample variable using BBKNN, which only permits correction over a single variable. Whilst the batch correction is not perfect, in the UMAPs we can observe strong mixing of the batch variables which include the sample ID (the corrected variable, n=70), the endoscopist who took the biopsy (n=31), the experimentalist who processed the sample for single-cell RNA-sequencing (n=8), the 10X bead batch (n=22), the 10x gem batch

(n=23) and the 10x chip batch (n=24).

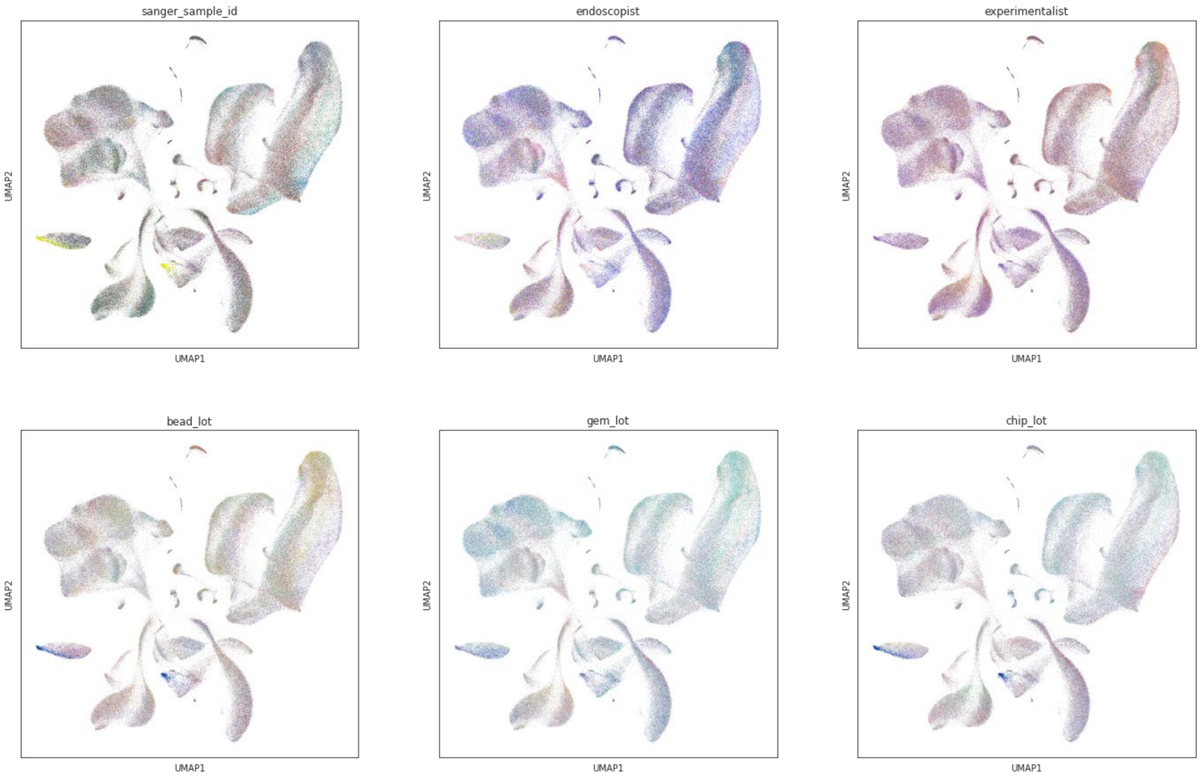

We also agree with the reviewer’s observation that comparison with public annotated data is valuable, to enable the reader to draw comparisons for similar cell types across studies. As such, we applied the Celltypist model trained on our atlasing cohort to the closest comparable dataset that is publicly available - Kong et al 2023. After subsetting their data for ileal samples, we applied our model and filtered to remove low-confidence assignments (Celltypist confidence <= 0.5). Overall, we observed a strong concordance between the annotation labels of our cell types and Kong et al.. With 44 / 56 of our annotated cell types mapping with >80% confidence to similar cells in Kong et al. Unsurprisingly, annotation is not uniformly identical. For example, of the 12 cell types with <80% cell typist confidence, 6/12 are populations of cells that exist in a continuum of differentiation (e.g. monocytes-macrophage and enterocytes). Reassuringly, we observe a discordant annotation in only 2/56 populations (T cells CD8+ tissue resident and Plasma B cell XBP1/CD38+++). Taken together, we are confident that our annotations, supported by the marker gene expression shown in Supplementary Figure 1, are robust. We present a full table of annotation comparisons in the supplementary data.

In response to the above comment we have added the following statement to the manuscript:

“The cell type classifier permitted the inclusion of a large number of cells that were confidently annotated despite not meeting the stringent quality control criteria applied to the atlasing cohort. Some of these cells, particularly those of the epithelial compartment, displayed signatures of cellular stress such as the expression of many mitochondrial genes (Fig. SX). To account for this mitochondrial gene expression level was included as a covariate in downstream differential gene expression analyses. The cell type classifier was also applied to a recently generated single-cell RNA-seq datasets of the ileum (Kong et al. 2023), with strong concordance observed between the cell type labels (Supp table S2).”

3. Differential Expression Analysis: The low consistency of DE genes between the discovery and replication cohorts should be further investigated. Could this be due to the analysis pipeline (e.g., potential high false discovery rates in single-cell DE analysis, see [https://www.nature.com/articles/s41467-021-25960-2 [nature.com]](https://www.nature.com/articles/s41467-021-25960-2))? Does the low consistency correlate with the number of cells per sample?

In response to this suggestion and the similar suggestion from reviewer 1, we re-analysed the full dataset using a pseudobulk approach. The findings were consistent with our linear mixed model approach with no improvement in concordance between cohorts. See the reviewer 1 response for a full summary of the approach and conclusions.

4. Pathway and Cell Linkage Analyses: Beyond DE genes, is the pathway analysis and cell linkage analysis consistent between the discovery and replication cohorts?

We note the reviewer’s observation that at present it is unclear if these analyses replicate between the discovery and replication cohorts and apologise for the lack of clarity in the text. For the cell type heritability analysis shown in Figure 5, all significantly enriched cell types were significant in the discovery, replication and full cohorts, independently. Similarly, in the gene-set enrichment analysis we only report pathways which were significant in both the Discovery and Replication cohort. The GSEA results reflected the DGE results in that whilst the majority of epithelial cell types showed strong

replication (>50% of significant terms replicate in both cohorts), the majority of immune and stromal cell types did not (<=50% of significant terms replicate in both cohorts).

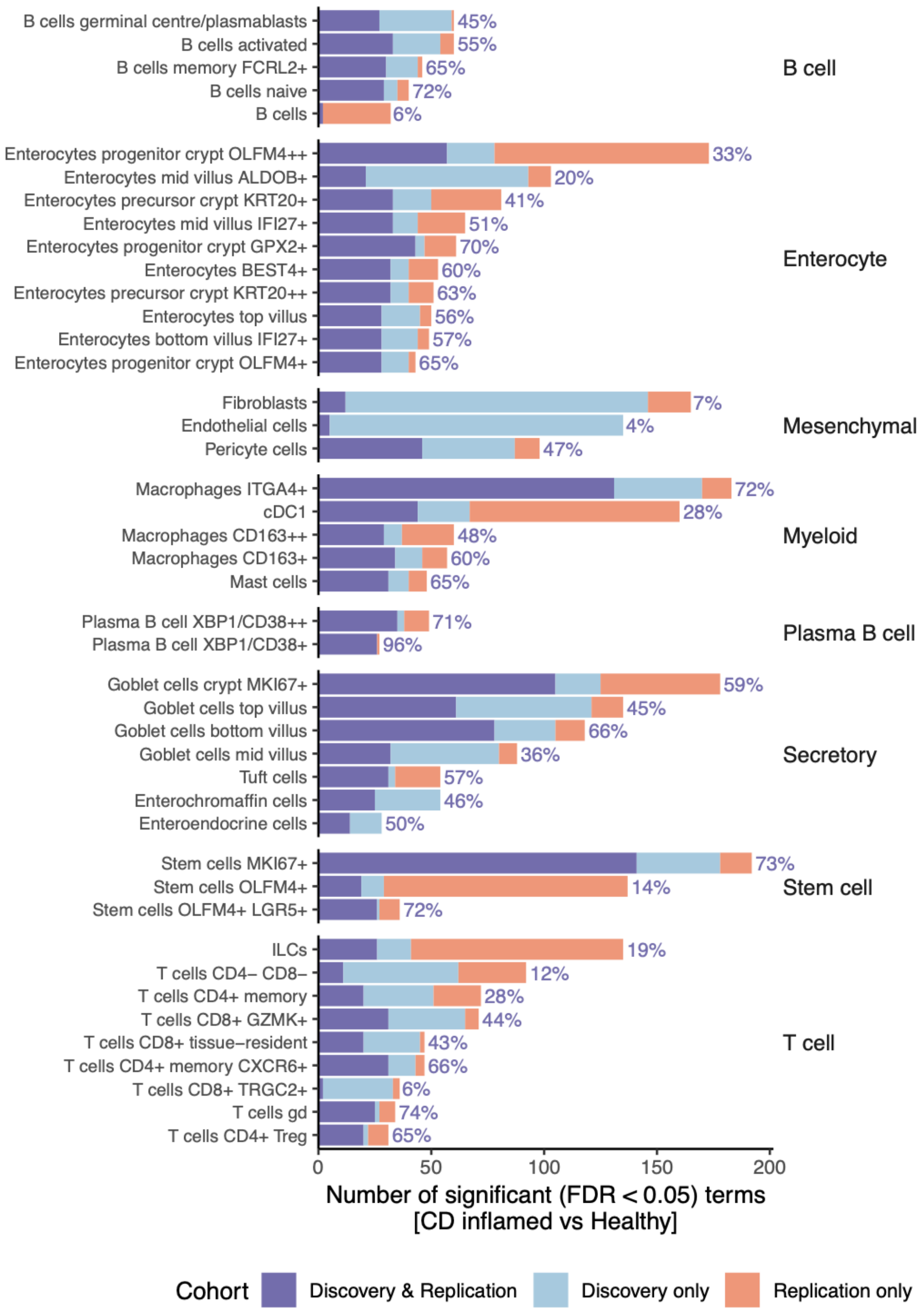

We have added the above figure as a supplementary figure and the following text to the manuscript:

"Gene sets also followed this trend with at least half of the significant terms (FDR < 5%) replicating in 13 out of the 20 epithelial cell types with at least one

enrichment, as opposed to 11 out of 21 immune cell types and no stromal cell types (Fig. S10).”

5. Gender Distribution: What is the male and female distribution within the samples? Have any gender-specific differences been identified?

We thank the reviewer for their suggestion and agree that it is a notable absence from our analyses despite the relevance of sex to many diseases, particularly immune disorders. As a result we performed a DGE comparison across sex however we did not find many genes significantly differentially expressed compared to the disease analyses (177 FDR < 0.05 in either cohort versus 4,855 for the primary disease analysis). We were aware that to make these DEGs a focus of the paper would require more downstream computational and functional analyses. Given the complexity of the paper as it is, we are reluctant to include and speculate on the results of these analyses in the paper (unless reviewer/editor think it necessary for publication). But we do include them in the shared outputs because we agree with the reviewer that sex differentiated analyses are important in order to distinguish factors driving sex differences in disease susceptibility and progression. We have also included the sex distribution between disease and between our two cohorts in our table 1 and supplementary table 2.

6. Transcription Factor Analysis: In addition to DE gene analysis, it is suggested that the authors perform TF–gene regulatory network analysis to identify TF regulators underlying cell state changes in CD samples.

While we agree this analysis is interesting, we are conscious that we already have a complex paper with many different analyses and are nervous about adding to this given this was not the main focus of our project. If the editors and reviewers feel this is required for publication we will perform the analysis, otherwise we will leave this as a future project to be performed by others.

7. The labels for Figure 3a/b are misassigned as Figure S3a/b in the manuscript.

This has been corrected now, thank you.

8. The IBDverse website is currently not functional. This should be fixed before the manuscript is published.

We have designed a website which will go live when the paper is published, along with links to the accompanying data. A preview of the website can be found here: https://ibdverse.webflow.io/.

Reviewer #2 (Remarks on code availability):

The provided code repository (https://github.com/andersonlab/sc_ti_atlas [github.com]) appears to be linked to the computational analyses in Krzak et al. 2023 rather than to this study. These issues should be addressed to ensure reproducibility and transparency.

We thank the reviewer for reminding us that we had neglected to update the github repository for the computational analyses. We have now updated the github repository detailing how to perform the analyses necessary to draw the conclusions we make in our manuscript.

Reviewer #2 (Remarks on figshare data availability):

The data are currently not available via Zenodo or the IBDverse website as listed in the manuscript.

The data is ready to be shared and will be deposited in Zenodo to be released upon publication. This will include the atlas anndata object, the full auto-annotated anndata object, the differential gene expression summary statistics, metadata data related to the patients and metadata related to the sample.
